# The Globalization of Traditional Medicine in Northern Peru: From Shamanism to Molecules

**DOI:** 10.1155/2013/291903

**Published:** 2013-12-28

**Authors:** Rainer W. Bussmann

**Affiliations:** William L. Brown Center, Missouri Botanical Garden, P.O. Box 299, St. Louis, MO 63166-0299, USA

## Abstract

Northern Peru represents the center of the Andean “health axis,” with roots going back to traditional practices of Cupisnique culture (1000 BC). For more than a decade of research, semistructured interviews were conducted with healers, collectors, and sellers of medicinal plants. In addition, bioassays were carried out to evaluate the efficacy and toxicity of plants found. Most of the 510 species encountered were native to Peru (83%). Fifty percent of the plants used in colonial times have disappeared from the pharmacopoeia. Market vendors specialized either on common and exotic plants, plants for common ailments, and plants only used by healers or on plants with magical purposes. Over 974 preparations with up to 29 different ingredients were used to treat 164 health conditions. Almost 65% of the medicinal plants were applied in these mixtures. Antibacterial activity was confirmed in most plants used for infections. Twenty-four percent of the aqueous extracts and 76% of the ethanolic extracts showed toxicity. Traditional preparation methods take this into account when choosing the appropriate solvent for the preparation of a remedy. The increasing demand for medicinal species did not increase the cultivation of medicinal plants. Most species are wild collected, causing doubts about the sustainability of trade.

## 1. Introduction

Traditional medicine is used globally and has a rapidly growing economic importance. In developing countries, traditional medicine is often the only accessible and affordable treatment available. In Uganda, for instance, the ratio of traditional practitioners to the population is between 1 : 200 and 1 : 400, while the availability of Western doctors is typically 1 : 20,000 or less. Moreover, doctors are mostly located in cities and other urban areas and are therefore inaccessible to rural populations. In Africa, up to 80% of the population uses Traditional Medicine as the primary healthcare system. In Latin America, the WHO Regional Office for the Americas (AMRO/PAHO) reports that 71% of the population in Chile and 40% of the population in Colombia have used Traditional Medicine. In many Asian countries, Traditional Medicine is widely used, even though Western medicine is often readily available. In Japan, 60–70% of allopathic doctors prescribe traditional medicines for their patients. In China, Traditional Medicine accounts for about 40% of all healthcare and is used to treat roughly 200 million patients annually. The number of visits to providers of complementary-alternative medicine (CAM) now exceeds by far the number of visits to all primary care physicians in the US [1–3].

Complementary-Alternative Medicine is becoming more and more popular in many developed countries. Forty-eight percent of the population in Australia, 70% in Canada, 42% in the US, 38% in Belgium, and 75% in France have used Complementary-Alternative Medicine at least once [4–6]. A survey of 610 Swiss doctors showed that 46% had used some form of CAM, mainly homeopathy and acupuncture [7]. In the United Kingdom, almost 40% of all general allopathic practitioners offer some form of CAM referral or access [8]. In the US, a national survey reported the use of at least 1 of 16 alternative therapies increased from 34% in 1990 to 42% in 1997 [9, 10].

The expenses for the use of Traditional and Complementary-Alternative Medicine are exponentially growing in many parts of the world. In Malaysia, an estimated US $500 million is spent annually on Traditional Medicine, compared to about US $300 million on allopathic medicine. The 1997 out-of-pocket Complementary-Alternative Medicine expenditure was estimated at US $2,700 million in the USA. In Australia, Canada, and the United Kingdom, annual Complementary-Alternative Medicine expenditure is estimated at US $80 million, US $2,400 million, and US $2300 million, respectively. The world market for herbal medicines based on traditional knowledge was estimated at US $60,000 million in the late 1990s [11]. A decade later, it was around US $60 billion [12] with estimates for 2015 at US $90 billion [13]. The sales of herbs and herbal nutritional supplements in the US increased to 101% between May 1996 and May 1998. The most popular herbal products included Ginseng (*Ginkgo biloba*), Garlic (*Allium sativum*), *Echinacea* spp., and St. John's wort (*Hypericum perforatum*) [11].

Traditional and complementary-alternative medicine are gaining more and more respect by national governments and health providers. Peru's National Program in Complementary Medicine and the Pan American Health Organization recently compared Complementary Medicine to allopathic Medicine in clinics and hospitals operating within the Peruvian Social Security System. A total of 339 patients—170 being treated with Complementary-Alternative Medicine and 169 with allopathic medicine—were followed for one year. Treatments for osteoarthritis; back pain; neurosis; asthma; peptic acid disease; tension and migraine headache; and obesity were analyzed. The results, with 95% significance, showed that the cost of using Complementary-Alternative Medicine was less than the cost of using Western therapy. In addition, for each of the criteria evaluated—clinical efficacy, user satisfaction, and future risk reduction—Complementary-Alternative Medicine's efficacy was higher than that of conventional treatments, including fewer side effects, higher perception of efficacy by both the patients and the clinics, and a 53–63% higher cost efficiency of Complementary-Alternative Medicine over that of conventional treatments for the selected conditions [14].

According to WHO [3], the most important challenges for Traditional Medicine/Complementary-Alternative Medicine for the next years are as follows.

- Research into safe and effective Traditional Medicine and Complementary Alternative Medicine treatments for diseases that represent the greatest burden, particularly among poorer populations.
- Recognition of the role of Traditional Medicine practitioners in providing healthcare in developing countries.
- Optimized and upgraded skills of Traditional Medicine practitioners in developing countries.
- Protection and preservation of the knowledge of indigenous Traditional Medicine.
- Sustainable cultivation of medicinal plants.
- Reliable information for consumers on the proper use of Traditional Medicine and Complementary-Alternative Medicine therapies and products.

Dr. Manuel Fernández, National Subdirector of Peru's Instituto Nacional de Medicina Tradicional (INMETRA), in the 90s delineates problems related to the production of phytopharmaceuticals in Peru.

- Lack of national policies.
- Absence of state and local policies that include medicinal plants.
- Lack of support by the state.
- Lack of support from the medical establishment.
- Ignorance of the benefits of the phytopharmaceutical industry.
- Limited human and technical resources.
- Lack of technical knowledge for the production of herbal products.
- Ignorance of methods and processes of quality control and standardization.
- Problems in obtaining quality materia prima in adequate quantities and predatory collecting.
- Absence of conservation policies implementing cultivation of herbals under best conditions.
- Limited research in ethnobotany, agrotechnology, pharmacy, and therapeutic validation.
- Lack of legal parameters for sanitary controls and commercialization of herbal products.
- Vested interests of the pharmaceutical industry minimizing the importance of herbs which are not the product of their own research and development.

Dr. Fernández also discusses a decreasing trend in Latin American consumption of medicinal products from 8% of global consumption in 1980 to 5% in 1990. He attributes this reduction to decreased government distribution of free medicines to the poor, concentrated wealth in a few hands, and increased poverty. Another factor is the fact that developed nations spend a much higher percentage of GDP on medicines (6–8%) than developing nations (1-2%) where it is estimated that 2/3s of medicines purchased are paid for by the patients. And per capita spending is much higher in developed nations compared with developing countries, for example, Japan: US $276; Germany: US $148; USA: US $128; Argentina: US $42; Uruguay US $40; Paraguay: US $18; Brazil: US $10.5; and Bolivia US $4. There are no figures for Peru, but it is estimated to be slightly above the amount for Bolivia. Overall, it is estimated that 50% of the population of Latin America has little or no access to medicines and that a large portion of these people use medicinal plants.

An innovative response to the challenges listed above has been developed by the Centro de Medicina Andina (CMA) founded in Cuzco in 1984 as an autonomous branch of the Instituto Pastoral Andina (IPA). Started by Catholic healthcare workers with extensive experience in Quechua communities, CMA's pragmatic methodology involves “mutual training” between health care professionals, community health promoters, *curanderos*, and midwives. For them, the rhetorical question is “Who knows all of the richness of Andean medicine better than the peasant himself, the specialist-practitioner of this medicine?”

Objectives of the Centro up to 1992 were “(1) Advance a health system favoring the majority of the community where Natural-Popular Medicine and modern medicine are complementary. (2) By means of study and application of Natural-Popular Medicine, create a scientific basis for its development.” Revised objectives since 1992 are “(1) Valorize and rescue Andean Medicine in order to contribute to better utilization and recognition within a system of alternative health available to a majority of the population. (2) Investigate, experiment, and disseminate the experiences and knowledge of Andean Medicine. (3) Encourage debate, exchange, and coordination between people and institutions working in the field of Natural-Popular Medicine. (4) Rescue Andean and “Andeanized” foods to improve food consumption.”

CMA's programs include the following. (1) *Education*: “Peasant to peasant” training of community health promoters and women's groups in cooperation with local universities and the Ministry of Health. (2) *Medicine and Medical Anthropology*: epidemiological and regional health-status diagnoses, evaluation of traditional therapies, and ethnography and publication of aspects of Andean culture and “cosmovision.” (3) *Ethnobotany and Phytotherapy*: collection and identification of 3,740 plants and development of an Herbarium and certified laboratory leading to the production and commercialization of six natural medicines.

Northern Peru represents the “health axis” of the old Central Andean cultural area stretching from Ecuador to Bolivia. The traditional use of medicinal plants in this region, which encompasses in particular the Departments of Piura, Lambayeque, La Libertad, Cajamarca, Amazonas, and San Martin, possibly dates back as far as to the first millennium B.C. (north coastal Cupisnique Culture) or at least to the Moche period (A.D. 100–800), with healing scenes and healers frequently depicted in ceramics.

Precedents for this study have been established by early colonial period chroniclers [15–18]; the plant collections (293 plants in crates 11 and 12 of 24) of Bishop Baltasar Jaime Martínez Compañón sent to the Palacio Real de Madrid along with cultural materials in 1789 under the title *Trujillo del Perú* in 9 illustrated volumes [19–21]; the travel journals of H. Ruiz from 1777 to 1788 [22]; the work of Italian naturalist Antonio Raimondi [23]; ethnoarchaeological analysis of the psychedelic San Pedro cactus [24]; *curandera *depictions in Moche ceramics [25], and research on the medicinal plants of Southern Ecuador [26–29].

## 2. Antecedents: Medicinal Plant Research and Traditional Medicine in Peru

Containing 78 of the 107 ecoregions of the world, in 1993, it was estimated that Peru had 17,143 taxa of spermatophytes in 2,485 genera and 224 families [30]. It is thought that only 60% of the Peruvian flora has been studied, with 1,400 species described as medicinal [31].

The importance of biodiversity for the Peruvian economy is enormous since 25% of all exports are living species, the uses of which are essential to local populations in terms of firewood, meat, lumber, medicinal plants, and many other products. Of particular importance are vegetal species, with 5,000 plants applied in 49 different uses. Of the 5,000 plants in use, some 4,400 are native; only 600 are introduced. The majority of useful native species are not cultivated; only 222 can be considered to be domesticated or semidomesticated [32].

Transculturation is resulting in an enormous loss of traditional knowledge of great value to the science and technology of Peru. The flora of the country represents 10% of the world's total, of which 30% is endemic. Peru is the fifth country in the world in number of plant species with known properties utilized by the population (4,400 species); it is the first in domesticated native species (182) [31].

In all Peruvian ethnic groups, plant knowledge is invaluable because it reinforces national identity and values, which are being lost in the complementary processes of modernization and globalization. In the current situation, the emerging recognition and incipient application of these resources and associated knowledge emphatically underscore the critical need for ethnobotanical research in light of the following facts.

- Absorption and devaluing of native culture due to modernization and globalization.
- At the same time, recuperation/revalorization of traditional knowledge of Peruvian flora.
- Emerging “first world” awareness of the therapeutic potential of medicinal plants.
- Recent ethnobotanical research by a growing number of Peruvian scholars [33].

In *Sinopsis histórica de la Etnobotánica en el Perú*, La Torre and Albán Castillo [34] outline the history of formal floristic studies in Peru starting in 1778 with the work of Hipólito Ruiz, José Pavón, and Joseph Dombey followed by Alexander von Humboldt and Aime Bonpland. Other early botanical explorers include Raimondi [23], Larco Herrera [35], Valdizan and Maldonado [36], Soukop [37], López et al. [38], Chavez (1977), de Ferreira [39, 40], and Duke and Vásquez [41]. However, it was John Harshberger who used the term ethnobotany for the first time in Peru while Juana Infantes actually established the discipline at the Universidad Nacional Mayor de San Marcos in 1945 [34].

Considerable progress has been made in the overall taxonomic treatment of the flora of Peru over the last few decades [36]. However, while the Amazon rainforests have received a great deal of scientific attention, the mountain forests and remote highland areas are still relatively unexplored. Until the late 1990s little work had been done on vegetation structure, ecology, and ethnobotany in the mountain forests and coastal areas of the north. In spite of the fact that this region is the core of what Peruvian anthropologist Lupe Camino [42] calls the “health axis” of Central Andean ethnomedicine, little ethnobotanical and ethnomedical research has been published on the rich flora found here.

Early ethnobotanically oriented studies focused mainly on the famous “magical” and “mind altering” flora of Peru. A first study on “cimora”—another vernacular name for the San Pedro cactus—dates back to the 1940's [43]. The first detailed study of a hallucinogen in Peru focused on the San Pedro cactus (*Echinopsis pachanoi*) [44, 45]. A variety of works including some on the “Daturas” (*Brugmansia* spp.) followed by [46–50] Coca (*Erythroxylum coca*) also attracted early scientific attention [51–56], as did the Amazonian Ayahuasca (*Banisteriopsis caapi*) [56–59]. Chiappe et al. [60] were the first to attempt an overview on the use of hallucinogens in shamanistic practices in Peru. More comprehensive accounts followed [61, 62].

In his classical study of Uña de Gato, Peru's leading advocate for Traditional Medicine and Founding Director of the Instituto Nacional de Medicina Tradicional del Ministerio de Salud, Fernando Cabieses [63] pointed out that the work of the Peruvian scholars Valdizán and Maldonado [36] was the pioneering effort in studying Traditional Medicine, leading to the emergence of medical anthropology nearly five decades later. In the interim, the botanical exploration of Peruvian flora and medicinal plants in particular included studies by Yacovleff and Larco-Herrera [64, 65]; Weberbauer [65]; Towle [66]; and Valdivia [67]. Most authors [35, 37, 40, 64–69] focused on Quechua herbalism of the Cuzco area. Other comprehensive studies were centered on the border region of Peru and Bolivia around Lake Titicaca [70–75] and the Amazon [41, 76–78] while Cabieses [79] wrote a major tract on Traditional Medicine. Detailed studies of Uña de Gato [63], Maca [80], and Sangre de Drago [81] were also carried out. Northern Peru, in contrast, was always in the shadow of more touristically important regions, attracting little scholarly attention until recently [82–87].

During the 1970s, the World Health Organization (WHO) was very proactive in advocating the integration of Traditional Medicine into public health programs in third world countries. This culminated in the Alma Ata Declaration of 1978, which proclaimed “health for all in the year 2000” [88]. Cabieses [63] described his struggles to implement the UN tenets in Peru, together with Seguin [89, 90] who advocated the incorporation of traditional folk psychotherapy into the modern institutional framework. In 1979, they organized the First World Congress of Traditional Medicine to build on the Alma Ata Declaration. As a result of coming up with such a “hair-brained” (*descabellada*) notion, they were nearly expelled from the prestigious Colegio Médico del Perú. In addition, Peru's Minister of Public Health declined the invitation to participate in the inaugural ceremonies. In spite of these setbacks, the congress was a resounding success with participants from 23 countries and sessions in Lima, Iquitos, and Cuzco. Few medical doctors attended, however. Peru's negative response to WHO initiatives contrasts markedly with that of Mexico where, in 1975, President Echeverría established the Mexican Institute for the Study of Medicinal Plants (IMEPLAM), inaugurating an era of official recognition of Traditional Medicine. Abigail Aguilar, Director of Mexico's National Herbarium, underscores the positive impact of WHO: “What happens is that no one studies what they have. Everyone devalues what they have, especially in countries like Mexico where we've been conquered and have had another culture imposed on us… So in the case of Mexico, there's a historic complex in which everything that smelled of plants was worth nothing. Academic medical researchers weren't very interested in that kind of resource… until they heard what the WHO said in the 1970s. That took hold in many countries, it definitely took hold here… because IMEPLAM was already in place” [91].

Building on the success of the first conference, in 1988, Dr. Cabieses presided over the second congress. This time things were different, with 4,000 participants from 41 countries. The Minister of Public Health, the Dean of the Colegio Médico, and the Mayor of Lima all participated in the inauguration ceremony, along with a long list of university authorities. Published acts of the congress included important contributions on the medicinal flora of Peru [92] and for the Southern Andes [73]. Subsequent publications of note included the southern highlands of Peru [69, 74] and the Peruvian Amazon region [41, 93].

While he was Director of the National Institute of Traditional Medicine, Cabieses was instrumental in coordinating a network of 16 ethnobotanical gardens in Peru, which included the cultivation of medicinal plants used by traditional herbalists [94]. He also facilitated scientific research on Traditional Medicine building a large database of herbs and monographs on 200 species of medicinal plants. In 2001, a new administration discontinued these innovative programs.

In his last years, from his position as Rector of the Universidad Cientifíca del Sur, Cabieses (2007) published his magnum opus on medicine in ancient Peru. He was also a strong critic of Peru's apathy regarding protection of its biocultural resources. In his book *Hoy y Ayer: Las Plantas Medicinales* [95], he reviewed the lamentable history of medicinal plant legislation in Peru throughout the 1990s. He pointed out that the nation followed the recommendations of the US Food and Drug Administration (US FDA), which he saw as totally inapplicable, a situation traceable to the “bicultural” nature of Peruvian society where the official scientific world view predominates over traditional “cosmovision.” This was occurring in spite of the fact that, since the 1970s, WHO has repeatedly formulated and refined guidelines for appropriate protection and sustainable development of medicinal plant resources and associated knowledge. Most of these recommendations were systematically ignored by the Peruvian Government. Bringing a personal perspective to bear on this matter, Cabieses (page 118) quoted a Peruvian Minister of Public Health who stated that medicinal plants and Traditional Medicine “aren't worth a thing” and that their study was “a waste of money and effort.” He ended his book (page 120) by contrasting the renewed European interest in medicinal plants with the Peruvian attitude

*“But here in Peru it's different. The lack of information and efficient research, education, and medical practice regarding the use of medicinal plants aggravate the fact that more than nine million human beings, a third of our population, in effect have as their only medical resource… the vegetal resources that surround them. The great unknown in our public health system is why so many physicians go to such lengths to exclude from their therapeutic activity the only resource that can control the suffering—not to mention the ailments—of such an important sector of our population.”*

In the first decade of the 2000s—although little had been done to protect and sustainably develop these valuable natural resources and associated knowledge—increasingly unfettered access was being granted to foreign pharmaceutical corporations. In 2004, a forum organized by the Peruvian Congress and the Sociedad Peruana de Derecho Ambiental (SPDA), an NGO dealing with environmental law, pointed out that foreign patent applications were pending or granted for 19 Peruvian plants and that the government was not making resources available to determine if the patents or claims met the requirements of patent law [96]. Adding insult to injury, on 28 March 2009, *Somos*, the news magazine of the prestigious daily *El Comercio *reported that, under the terms of the Peruvian-North American Free Trade Agreement, claims by American pharmaceutical companies were to be granted “exclusive protection” for alleged “new products” regardless of whether or not they qualified or had prior licenses or patents [97]. Seguin and Cabieses would turn in their graves!

A classic example of one hand not knowing what the other is doing was revealed on 16 July 2009 when Portillo [98] reported that Peru had denied patents to companies from France, Japan, South Korea, and the US on the grounds that their products were developed using traditional knowledge. The denials emanated from the Peruvian National Commission Against Biopiracy advocated in the Peruvian Congressional Forum of 2004. However, the Portillo article ended with a quote from Michel Pimbert of the International Institute for Environment and Development: “It would be naïve to think that national governments would automatically share benefits with local communities when biopiracy is prevented or compensation obtained.” That said, in 2009, the public health section of Peru's social security system (EsSalud) inaugurated a pilot program to prescribe medicinal plants in three of its centers for Complementary Medicine in Lima, Arequipa, and Trujillo [99].

## 3. Issues in the Globalization of Traditional Medicine

Moran et al. [100] trace the emergence of biodiversity prospecting. On 5 June 1992, in order to alleviate the loss of earth's flora and fauna, the Convention on Biological Diversity (CBD) was inaugurated at the UN Earth Summit in Rio de Janeiro, Brazil. CBD objectives are (1) conservation of biodiversity, (2) sustainable use of components of biodiversity, and (3) equitable sharing of benefits derived from commercial use of genetic resources.

For biodiversity-rich developing countries, the most critical element in the CBD is sovereignty over bioresources by nation states, since the treaty recognizes their right to regulate and charge outsiders for access to their biodiversity. The sovereignty component is meant to replace the “common heritage” paradigm, which provides unrestricted access to biological resources. Ideally this paradigm shift is supposed to balance the way in which all involved interest groups can gain from biodiversity use by recognizing the economic, sociocultural, and environmental values of bioresources and the cost of their preservation.

In the time since the CBD was initiated, few of the 178 signatory nations have introduced legislation requiring benefit sharing for outside commercial access to their national bioresources, although some suggestions for implementation of the CBD have been brought forward [101, 102]. Despite the lukewarm response to the CBD by nation states, the global shift in awareness concerning tropical deforestation provided an opportunity for ethnobotanists to assert that everyone has an interest in preserving rainforests because they might contain compounds that could cure cancer, HIV-AIDS, and other diseases [103–107]. In addition, income derived from the marketing of traditional medicinal knowledge was seen as an instrument to alleviate poverty and to finance conservation efforts [108–110]. Within a few years, however, for its critics, ethnobotany—initially seen as instrument that could help to salvage declining traditional knowledge and biodiversity—had simply become an instrument of theft and “biopiracy.”

In his book *Who Owns Native Culture*? anthropologist Brown [107] has a chapter entitled “The Ethnobotany Blues” which documents high-profile projects launched in Africa and Latin America in the early 1990s. They were organized under the U.S. initiative known as the International Cooperative Biodiversity Groups (ICBG), administered by the Fogarty International Center for Advanced Study in Health Sciences, part of the National Institutes of Health (NIH), with additional funding from the National Science Foundation (NSF) and the U.S. Agency for International Development (USAID). Projects involved partnerships between American and host-country scientists as well as major drug companies, including Monsanto, Bristol-Myers Squibb, and American Cyanamid. Brown describes ICBG-Peru's troubled relationship between the Aguaruna of the Peruvian Amazon and Washington University (St. Louis), criticizing “paternalistic interventions that leave native peoples on the margins of decision-making and profit-taking” (page 114). In Mexico, he documents how ICBG-Maya was shut down by an indigenous healers' organization and their activist allies on the grounds that it was an effort to steal native knowledge and resources. And he traces the failure of Shaman Pharmaceuticals, a California company which folded in 1999, in trying to adapt ethnobotanical bioprospecting to the “magic-bullet” paradigm of the pharmaceutical industry.

In the late 1990s, anthropologist Hayden [91] conducted an ethnography of an ICBG bioprospecting agreement inaugurated in 1993 between the University of Arizona and its pharmaceutical partners (whose contribution was a discount on the use of their equipment!) and a team of plant researchers at Mexico's National Autonomous University (UNAM) headed by ethnobotanist Robert Bye. Under the agreement, UNAM researchers sent extracts of Mexican medicinal plants to the US in exchange for research funds and promises of a percentage of royalties 10 to 20 years in the future—should a drug result from the collaboration. The project was also designed to collect ethnobotanical knowledge and to direct some royalties back to source communities. It concluded in 2003 when UNAM opted out of a second renewal.

Hayden elucidates the complex issues that emerged during the project, in particular the paradoxical effects of NIH's advocacy of benefit-sharing according to the neoliberal paradigm of bioprospecting. For NIH, this meant that field researchers were supposed to sign contracts with each individual supplier of plants. Suppliers—and, by implication, their communities—were presumed to be “authors” and “stewards” of resources as well as future benefit recipients. For UNAM ethnobotanists, drawing on a well-established research methodology, this meant collecting initial plant species from urban marketplaces and rural roadsides, a major disruption of a fundamental bioprospecting assumption that plants and knowledge “come with” clearly identified local stewards, authors, and claimants.

In stark contrast with the ICBG approach, there is the Mexican Institute for Social Security (IMSS) model put into practice at its Southern Center for Biomedical Research (CIBIS) in Cuernavaca and focused on the production of herbal medicines. On 20 February 1997, Hayden [91] interviewed Miguel Antinori, a prominent CIBIS official who denigrated bioprospecting agreements for using Mexico's chemists as “cheap labor” and for sending extracts abroad for “more sophisticated” work. Further, he added, “It's hard to see an assertion of [Mexican] national identity in these contexts—up north, they just see Mexico as a source of raw material and certainly not as research partners or collaborators. Why do not they locate more of the development part here? Because they do not trust Mexican science.”

Former Shaman Pharmaceuticals scientists Moran et al. [100] discuss the irony in the situations described above, indicating that the majority of the biotech industry is not involved in bioprospecting, since most companies favor the use of cheaper and faster synthetic technologies over the complex process involved in exploring for natural products. Nonetheless, biotechnology spawns ethical, social, and legal debates at the margins of pharmaceutical bioprospecting, including the collaboration between big business and big science, the ethics of genetic engineering, and the patentability of life forms as well as ideas about genetics and racism, culture, and ethnicity. However, it is significant to note that, since the inauguration of the CBD, no pharmaceutical bioprospecting product developed by using traditional knowledge has generated an economic profit. (But this does not mean that pharmaceutical companies do not try to impede or coopt efforts to get natural plant products to market.) Also, only a small number of bioprospecting research expeditions begin by using ethnobotany as a discovery methodology, with the work soon evolving into economic botany as the laboratory focus shifts to the plant's chemistry, biological activity, and pharmacology/toxicology. During drug discovery, active chemical components are isolated, often modified, and patented. Patented information then becomes a commodity in itself.

Peruvian pharmaceutical researcher Angulo [111] discusses new approaches to research on medicinal plants contrasting Western and Eastern methodologies. For example, while the West does not value popular wisdom and usage developed over centuries by local cultures, the east uses this knowledge as a paradigmatic base for its model of science. While the West has exclusively followed the Cartesian model of scientific skepticism, Eastern pragmatism, building on tradition, has formalized usage and then applied the methods of modern science. While the West has ignored traditional knowledge in designing artificial studies that isolate chemical components and evaluate their toxicity and bioactivity to later take finished products into clinical settings, the East has followed an inverse strategy, that is, valuing traditional knowledge by applying original remedies and therapies in the medical clinic and then subjecting those that work to biochemical research and development. While the West followed a basic research paradigm of random screening, component analysis, and synthesis, the East recognized the holistic action of herbal medicines in seeking ways to industrialize them. As a result of the foregoing factors, Western science has developed economic botany, which uses a methodology of chemical taxonomy based on the assumption that only by knowing the chemistry of plants we can discover their active principles and bioactivity. This has led to the current emphasis on synthetic chemistry for the development of modern medicines.

Angulo (page 363) points out that, by uncritically following the Western model for biochemical research promoted by large European and American pharmaceutical corporations, Peru has acquiesced to the notion that countries like Peru and Mexico lack the technical and economic resources necessary to compete with foreign consortiums. As a result, these countries, for the most part, have denigrated their own indigenous knowledge and neglected the development of viable national research programs in ethnobotany and ethnopharmacology. Joining Elisabetsky and Castilhos [104], Angulo suggests that

*“Traditional medicine should be the basis for the development of drugs, given that it includes the knowledge of the therapeutic value of local flora. Thus, knowledge of the practices of Traditional Medicine plays a crucial role in the selection of species to subsequently be considered as potential sources of universally applicable drugs. Elisabetsky and Castilhos concludes that the interaction between anthropology and ethnopharmacology is the basis on which should be developed the holistic investigation of medicinal plants in particular and healthcare in general.”*

We would only add that applied research on natural plant remedies should also be on the national agendas of Peru and neighboring republics.

Manek and Lettington [112] point out that by focusing on indigenous knowledge as it relates to the environment, the convention on biological diversity managed to sidestep some of the more politically charged aspects of the intellectual property rights (IPR) issue. The greatest impact on concerns over indigenous and local community rights can be traced to the mercurial rise of biotechnology on the international trade front and the 1995 version of the World Trade Organization (WTO) Agreement on Trade Related Aspects of Intellectual Property Rights (TRIPS). These two factors have created a large potential market for indigenous and local knowledge and resources, while at the same time raising concerns about the risk that these resources will be misappropriated. Thus, this knowledge is receiving increasing international attention in terms of its relationship to human rights as well as its relevance to modern science. The situation has created opposing pressures calling for the rights of local and indigenous peoples on the one hand and further exploitation of their knowledge on the other. Moran et al. [100], Manek and Lettington [112], and Greaves [113] indicate that the biggest problem with the orthodox intellectual property system is its focus on material aspects of knowledge at the expense of the cultural. They advocate recognition of alternative worldviews in the formulation of new indigenous knowledge rights that are localized, relevant, pertinent, and effective.

In their article in *Cultural Survival Quarterly,* Bannister and Barrett [114] contend that bioprospecting is a form of economic botany that can run contrary to the ethnobotanical objectives of protecting biological and cultural diversity. The economic focus of this activity highlights issues concerning indigenous rights, cultural knowledge, and traditional resources—areas in which current intellectual property protection regimes are inadequate and inappropriate. However, indigenous communities are increasingly forced to employ intellectual property rights to protect these resources. Protection issues ought to be addressed well before the point at which employing intellectual property mechanisms seems to be the only alternative. Significant control lies at the point of decision about publication and dissemination of knowledge to the wider community, which raises important questions about facilitating the appropriation of cultural knowledge. The authors (page 10) advocate a more “precautionary” approach to ethnobotanical inquiry in assisting indigenous communities in protecting their cultural heritage and intellectual property rights.

Probably the major concern in many traditional communities is that their spiritual legacies will be profaned by a secularized and consumer-driven outside world. Often, however, legitimate economic considerations also play a role in the defensive reactions of these societies to the well-intended but naïve desire of the academic world to place its findings in the public domain. Greaves et al. [113, 114] have warned that the downside in this approach is that a “colonializing archive” can become easily “mined” for clues in the search for new drugs without the inconvenience of fieldwork or inclusion of source communities in the benefits derived from products resulting from research.

However, despite acknowledging genuine concerns about neocolonialism and biopiracy, we would submit that each situation has to be considered on its own merits, especially with regard to its specific cultural context. A first step in the evaluation process should involve the important distinction between “indigenous peoples” and “local communities” [100]. The latter for the most part is farmers who speak the national language, practice the majority religion, and identify with the nation state, especially with regard to their socioeconomic aspirations, whereas the former tends to be tribal and/or ethnic minorities, who seek collective rights and self-determination for their biological and cultural resources. Although it is often the case that in both communities traditional knowledge and resources are undocumented and in danger of disappearing, this danger tends to be more pressing in local communities as their members continue to adapt to privatization and globalization. In cases such as this successful ethnobotanical intervention, is required a methodology that combines “salvage ethnography” with “rapid assessment”. This is the methodology that we initially applied in Peru, motivated by our prior experience in Southern Ecuador where traditional knowledge of medicinal plants similar to those found in Northern Peru is diminishing at an alarming rate. However, with our database firmly established as a research vehicle, we can now turn our attention to facilitating proactive issues of education, conservation, and sustainable development of natural plant products.

India provides a positive example of the proactive application of this approach. Taking advantage of the “novelty” criterion in international patent law, with regard to the documentation of Ayurvedic and other traditional medicine, millennial Sanskrit texts as well as modern publications are included in a traditional knowledge database, which is subsequently provided to patent agencies. The expectation is that, by placing the knowledge about long-term cultural precedents for traditional uses in the public domain, this research will prove that contemporary patent applications derived from local medicinal knowledge lack originality that is that they are not “novel” enough to qualify as inventions warranting protection under international patent law and are thus not patentable.

Fortunately, in 2002, with the support of the International Phyto-Genetic Resource Institute (Rome, Italy), Peru promulgated Law 27811 for the protection of the collective knowledge of indigenous peoples related to biological resources. Article 17 of the law establishes a National Public Register to include collective knowledge that is in the public domain. This register is administered by National Institute of Competitive Defense and Intellectual Property (INDECOPI), with the obligation to send the information recorded to principal patent offices around the world, a protective defense mechanism intended to prevent the granting of patents which do not meet the criteria of novelty and degree of inventiveness [96, 115].

As noted earlier, Peru has also activated the Peruvian National Commission Against Biopiracy. In the Congressional Forum of 2004, which led to the formation of the Commission, a number of important issues were addressed, including intellectual property, the high protein cereal Quinoa and biopiracy, passage of the law for the protection of Peruvian biodiversity and the collective knowledge of indigenous peoples, and efforts to annul the US patent for the virility stimulant Maca as well as suggestions for combating biopiracy [116]. Briefly noted was the issue of genetically modified foods, anticipated as a concern that was likely to emerge with approval of a free trade agreement with the US [116]. When the Commission was legally mandated, later in 2004, 19 plant claims were slated for review. By 2010, claims for 69 plants were being researched, 17 cases of biopiracy had been identified, and seven (from France, Japan, and South Korea) had been successfully blocked. One hopes that in all these deliberations the following remarks by forum panelist Agurto [117] will be borne in mind:

*“The problem underlying biopiracy is the open recognition of the rights of the indigenous peoples and communities. Many times they have been excluded and marginalized from the politics of Government. Even today we encounter members of Congress who are either unaware of the existence of indigenous peoples or who do not recognize their rights. It is impossible to speak of biopiracy if we do not defend the holders of many genetic resources, those who have achieved the domestication, knowledge, and technology to utilize biodiversity in a sustainable fashion. They are also the holders of the right to prior informed consent, a fundamental right to know the objectives of the exploration and exploitation of their resources and traditional knowledge and the consequences or potential benefits that can come with industrial, commercial or scientific uses.”*

Spanish anthropologist Abad [118] concludes in her book *Ethnocide and Resistance in the Peruvian Amazon* that foreign and domestic development policies contribute to the marginalization of indigenous people:

*“Underdeveloped, developing, Third World, North-South…, perhaps the language has been changing in these times and the terminology has been adapting itself to partially new habits, but the unequal, hierarchical reality remains the same, given that those who exercise power continue to be the same. International assistance also keeps promoting unequal development between peoples.”*

## 4. Biodiversity Conservation and Traditional Medicine

A policy report, *Biodiversity, Traditional Knowledge and Community Health: Strengthening Linkages,* published by the United Nations University, Institute of Advanced Studies in Yokohama, Japan, addresses many of the issues discussed above [119]. Building on the WHO Alma Ata Declaration of 1978 related to Traditional Medicine and primary health care, the UN Convention on Biological Diversity of 1992, and the UN's Middle Development Goals (MDGs) of 2011, this document shows that links between Traditional Medicine and biodiversity are strengthened by three processes: (1) a medical approach involving national efforts to integrate Traditional Medicine into institutional healthcare delivery which includes challenges related to safety, quality, efficacy, access, and regulation; (2) a market-oriented approach focused on drug development or tourism promotion focused on biomedical products and services as marketable commodities; and (3) a community-focused approach activated by civil society organizations focused on conservation implemented through a grassroots mobilization process involving health professionals, botanists, conservationists, and community activists.

The community-based approach shows allegiance to the Alma Ata primary health care model. Examples include the barefoot doctors strategy in China and the social health activist programs in India. Given the centrality of biodiversity in human lives, there is still a need to develop sustainable strategies for health maintenance combined with conservation of biological resources linked to local knowledge and practices. This is relevant even in developed countries where there is an increasing demand for alternative and complementary medicine.

At the beginning of the UNU report in a “Message from the Director,” Govindan Parayil (page 6) assesses progress towards the CBD agenda of a global development path that is sustainable, equitable, environmentally just, and economically rewarding. He sees that the prognosis is not encouraging. Progress has been made, but we still are falling far short in even sustaining current levels of well-being. “Negative environmental trends continue to be exacerbated by human interventions—primarily led by a model of unsustainable and conspicuous consumption.” He adds: “The extraordinary emphasis on developing produced capital appears to have overwhelmed all other aspects of natural capital required for our well-being.”

On the positive side, Parayil notes increased awareness of the gap between planning and implementation. Welcome signs of change include “increasing resolve to align production activities with environmental and equity considerations” as well as “efforts aimed at reforming global institutional structures to create more synergies and effective implementation of relevant policies.” He concludes

*“Current accepted standards of practice and business norms must be re-oriented to include a more consultative policy setting with all major actor representatives. [This] would require designing regulations that acknowledge the need for balance among all forms of capital, and incentives that provide equitable access to resources and services.”*

The UNU policy report documents 30 successful community-based projects from around the world. Despite their success in finding workable solutions to meet conservation and primary health care needs, the scale of operation of these programs has not been enhanced or expanded. This is due to a number of factors listed in the report, some of which include the following.

- There is a clear need to include ecological, conservation, and sociocultural factors in goal-setting related to health and development programs.
- High external dependency, especially in pharmaceuticals and medical technologies, disincentivizes local innovations in Traditional Medicine and healthcare.
- Through a top-down health care approach, societies have organized themselves to be more disease-centric (with supporting institutions, research, industry, government departments, strategies, and programs) than wellness-centric. A paradigm shift in the mind set as well as in systems and structures to wellness (prevention/promotion) is a challenge, yet essential. For this to occur, internalization (not mere awareness) and implementation are essential.
- Traditional health promotion and related conservation schemes focused chiefly on medicinal plants have been seen more as avenues for economic development and hence expected to become self-supporting… To realize self-sufficiency, costs of delivery of various related services from resource collection to distribution and infrastructure to identify and support healers need to be thought out comprehensively. There is a critical need for innovative approaches for funding mechanisms in this area.
- At the policy level, there appears to be a tendency towards nonrealistic target setting. Implementation mechanisms for such targets still rely primarily on formal mainstream processes such as modern infrastructure and trained doctors, while including and appropriately training specialists outside the “modern” realm of training, especially at the community health worker level, might have hastened the processes to achieve various goals. A reflexive learning approach to development is especially important in this context where no single knowledge system has sufficient conceptual, theoretical, or practical authority in addressing health challenges.
- While attempts to document and protect traditional medical knowledge in searchable and other inventories are important in terms of defensively protecting such knowledge from misappropriation, efforts to use such knowledge to augment community health are still insufficient. Attempts to open such inventories for research purposes still play into mainstream drug development processes—more than local healthcare. This stymies efforts to use and expand such initiatives to provide better community and public healthcare.
- High erosion of traditional knowledge and lack of perceived support for traditional health practitioners have evidently led to a decrease in the receptivity to and transfer of all aspects of such knowledge between generations. It has been observed that in cases where the knowledge system is perceived to bring in recognition and supplemental income, younger generations are more keen to learn, develop, and sustain them.
- We often see that the dominant education and research systems tend to enhance knowledge and technologies using universal standards, without much attention to the capacities and needs of specific regions or populations resulting in a dearth of comprehensive theoretical approaches to assessing traditional knowledge which is believed to be key to the disregard of traditional cultures This then calls for the design and implementation of culturally appropriate pedagogical methods with an intercultural inclination and transdisciplinary approach and their integration into formal and informal learning processes.
- There appears to be a clear need for designing a radical and innovative approach to integrate Traditional Medicine into mainstream health systems. This would further require full institutional backing from various related governmental and nongovernmental agencies that link supply chains of medicinal resources with health practitioners and consumers with the highest standards of quality, safety, and efficacy.

With regard to a plan of action, this policy paper advocates the use of integrated rapid assessment protocols similar to those used in some of the case studies outlined in the report—duly adapted to local cultural and environmental circumstances. It provides an assessment framework and the following “potential strategies.”

- Assessment methods to inventory resources and knowledge used in healthcare.
- Knowledge validation, generation, and use.
- Capacity building for different stakeholders.
- Cross-learning between different knowledge systems.
- Mechanisms to protect traditional resources and knowledge.
- Linking with economic development objectives.
- Expansion of partnerships with different stakeholders.
- Effective communication strategies.
- Synergizing community initiatives with civil society organizations and policy processes in identifying critical areas of engagement.

Complementing the positive examples from the UN University-Yokohama report is the lesson learned from a failed project in Northern India which sought to develop a medicinal plant value chain between local Himalayan farmers and a Dutch company (Ayurveda Health) in a project undertaken by The Royal Tropical Institute (KIT) and the Center for Sustainable Development (CSD) of The Netherlands in cooperation with local government agencies [120]. The authors point out that worldwide medicinal plants are being depleted at a rapid pace due to large-scale, unsustainable collection from natural habitats. Conservation of these species is critical for four reasons: (1) they are a source of natural ingredients used by the manufacturers of modern pharmaceuticals resulting in a large and increasing demand [121–123]; (2) medicinal plants form the basis of homeopathy and traditional medicines, and, along with traditional knowledge, are crucial for traditional healers, who play a vital role in the lives of poor people and their animals in developing countries [2]; (3) the collection and marketing of medicinal plants are a valuable source of livelihood for large numbers of poor people in developing countries; and (4) medicinal plants are an essential component of biological diversity and conservation.

Regarding lessons learned, three reasons are given for the project's lack of success: (1) poor quality planting material supplied to farmers resulting in high mortality of plants; (2) too many uncoordinated farmers planting uneconomic plots on marginal land which resulted in low upkeep motivation and unrealistic expectations that were not realized; and (3) poor understanding of local farming dynamics and the emergence of a successful alternative cash crop. These are factors that should be evaluated in any efforts to build a successful supply chain for CMC-EsSalud-Trujillo.

## 5. Two Decades of Traditional Medicine Research in Northern Peru

Work up to 2012—besides developing a database of 510 medicinal plants [124–126] and 974 remedies of mixtures [127]—has demonstrated that herbal commerce in Peru is a major economic resource [128], which, although used alongside modern pharmaceutical products, is showing signs of diminished popular knowledge of applications [129, 130]. Laboratory research on most of the database has ranged from minimum inhibition concentrations [131] to toxicity screening [132] as well as bioassays to determine antibacterial activity [133–137] and phytochemical analysis [138, 139] with more focused analyses of herbal treatments for acne [133] and malaria [140]. Other studies have sought to identify Ulluchu, a ceremonial plant of the pre-Hispanic Moche culture [141] as well as surveying colonial sources of medicinal plants in Northern Peru and Southern Ecuador [126]. An ethnography of peasant herbalists which documented aspects of the market supply chain showed that suppliers are not adequately remunerated and revealed threats posed by lack of conservation measures and overharvesting [142, 143] criticized the scientific reductionism of laboratory research in attempting to appropriately verify traditional remedies. Anthropological studies of traditional *curanderos* and their curing altars (*mesas*) include articles by Sharon et al. [144]; Sharon and Gálvez [145]; Sharon et al. [146]; and Glass-Coffin et al. [25].

It is worth noting that, during the decade that we have been working in the field and the laboratory, there has been a sea change in attitudes and perceptions of Traditional Medicine [147–178]. In Trujillo, Lima, and Arequipa, a pilot program prescribing medicinal plants, scientifically validated by WHO/PAHO, has been initiated by EsSalud's National Program for Complementary Medicine, an initiative that begun in 1999 with three centers which has grown to 26 to date [149, 150]. In Trujillo, the Missouri Botanical Garden's Sacred Seeds program has started an herbal garden and educational outreach program at the site museum of the pre-Hispanic Chimú city of Chan Chan. In Huamachuco, a program of ethnobotany and conservation manifest in community gardens and seed banks of medicinal and food plants is slowly emerging through collaboration between three local peasant communities, the Beneficencia Publica and regional hospital, MBG's Sacred Seeds program, MHIRT, and the Peace Corps. Future work will involve developing a supply chain between Huamachuco and CCM-Trujillo with scientific validation by MBG, UB (SUNY), the Biotransformation and Natural Products Laboratory at UNT, and the Interdisciplinary Research Group at UPAO as coordinated by MHIRT and MBG.

### 5.1. Plant Nomenclature in Northern Peru

For the last decade, the nomenclature of plant families, genera, and species registered in Northern Peru followed the *Catalogue of the Flowering Plants and Gymnosperms of Peru* [30]. Species were identified using the available volumes of the *Flora of Peru* [151] as well as work on the flora of Ecuador and Bolivia [152–155] and reference material in the herbaria HUT and HAO.

The naming of plant species follows three general patterns. Plant names already used by original indigenous populations are often maintained, although slightly modified. Plants similar to species already known, or with similar habitus, often receive the same name (transposition). In other cases, completely new names are created (neology) [156].

The vernacular names of the plants used in Northern Peru reflect the historical development of plant use in the region. Introduced species (e.g., *Apium graveolens*—Apio, *Foeniculum vulgare*—Hinojo) and native species similar to species found in Spain (e.g., *Adiantum concinnum*—Culantrillo, *Matricaria frigidum*—Manzanilla), as well as species growing mostly in the coastal regions of the area (e.g., *Alternanthera porrigens*—Sanguinaria) are often addressed with names derived from Spanish roots. Plants from the mountain forests and especially the Andean highlands or the Amazon are often known by their Quechua names (e.g., *Pellaea ternifolia*—Cuti Cuti, *Amaranthus caudatus*—Quihuicha, and *Banisteriopsis caapi*—Ayahuasca), and a few plant names can be traced back to Mochica (the original indigenous language spoken at the coast of Northern Peru) roots (e.g., *Nectandra* spp.—Espingo) [157]. Van den Eynden et al. [156] observed similar patterns in Southern Ecuador, although her study focused only on edible species. Nine hundred thirty-eight vernacular names were recorded for 510 plant species. About one-third of all names represented Quechua names or had Mochica roots, while 66.5% of all names were of Spanish origin or at least had Spanish components. In comparison, 41% of the vernacular names of edible plants in Southern Ecuador were found to be of Spanish origin. More than half of the indigenous species carried only one vernacular name, with the remaining species carrying a variety of indigenous names, often derived from the same root. In comparison, almost 75% of the introductions were known by one name only. The slight differences in plant names indicate that the species have been used in the region for a long time, and that their names reflect small variations in the local dialects.

### 5.2. Two Decades of Ethnobotany in Northern Peru and Southern Ecuador

Ethnobotanical data were collected from plant sellers while purchasing plant materials in local markets (mostly Mercado Mayorista and Mercado Hermelinda in Trujillo and Mercado Moshoqueque and Mercado Modelo in Chiclayo); by accompanying local healers (*curanderos*) to the markets when they purchased plants for curing sessions and into the field when they were harvesting. In addition, plants were collected by the project members in the field, and—together with the material purchased in the markets—were taken to the homes of *curanderos* to discuss the plants' healing properties, applications, harvesting methodology, and origins. At the *curanderos'* homes, the authors also observed the preparation of remedies and participated in healing rituals. Plant uses were discussed in detail with informants, after seeking prior informed consent from each respondent. Following a semistructured interview technique, respondents were asked to provide detailed information about the vernacular plant name in Spanish or Quechua; plant properties (hot/cold); harvesting region; ailments for which a plant was used; best harvesting time and season; plant parts used as well as mode of preparation and application; and specific instructions for the preparation of remedies, including the addition of other plant species. All interviews were carried out in Spanish, with at least one of the authors present. Both authors are fluent in Spanish, and no interpreter was needed to conduct the interviews. Data on plant species, families, vernacular names, plant parts used, traditional uses, and modalities of use were recorded.

Many of the species reported from Northern Peru are widely known by *curanderos* and herb vendors as well as the general population of the region and are employed for a large number of medical conditions. One hundred fifty to two hundred plant species, including most of the introductions, are commonly sold in the local markets [126]. Rare indigenous species were either collected by the healers themselves or are ordered from special collectors or herb vendors. The same plants were frequently used by a variety of healers for the same purposes, with only slight variations in recipes. However, different healers might give preference to different species for the treatment of the same medical condition. All species found were well known to the healers and herb vendors involved in the study, even if they themselves did not use or sell the species in question. Many species were often easily recognized by their vernacular names by other members of the population. This indicates that these remedies have been in use for a long time by many people. The use of some species, most prominently San Pedro (*Echinopsis pachanoi*), Maichil (*Thevetia peruviana*), and Ishpingo (various species of *Nectandra*), can be traced back to the Moche culture (AD 100–800). Representations of these plants are frequently found on Moche ceramics, and the remains of some were found in a variety of burials of high-ranking individuals of the Moche elite, for example, the tomb of the Lord of Sipán [157].

### 5.3. Medicinal Uses

Five hundred and ten plants with medicinal properties were registered in Northern Peru. The same species was often used for various medical conditions and applied in different ways for the same condition. For example, nervous disorders might be treated using different parts of a plant in different applications, for example, topical (as a Poultice or Bath), oral (ingestion of plant extracts), and by supplying the patient with a *seguro*, a bottle filled with herbs and perfumes, which serves as a protective charm. Two thousand four hundred ninety-nine different uses were registered for the 510 species encountered. In the following, the total number of uses/applications and the number of species used are given, rather than only the number of plant species used to treat a condition, in order to emphasize the importance of the treatment of specific conditions.

The highest number of species (207, 40.4%) is used for the treatment of “magical” ailments, with 682 (27.3%) of all conditions. Respiratory problems (95 species, 18.5%) were mentioned as 233 (9.3%) of all uses; 98 species (19.1%) are used to treat psychosomatic and nervous system problems, with 176 applications (7%). Kidney and Urinary tract disorders are treated with 85 species (16.6%) for 111 conditions (4.4%). Rheumatic and arthritic symptoms are mentioned in 103 uses (4.1%) with 45 species (8.8%) used for treatment. Infections of female organs are treated with 66 species (12.9%) and comprised 100 (4.4%) of all conditions.

Treatments are most often performed in the homes of the individual healers, who normally have their *mesas* (healing altars) set up in their backyards. Healers also treat patients at altars and consultation chambers (*consultorios*) in their homes, at sacred sites in the countryside, or at sacred lagoons high in the mountains. Healing altars (*mesas*) bearing a large number of power objects are often employed. A curing ceremony normally involves purification of the patient by orally spraying blessed and enchanted herbal extracts on the whole body to fend off evil spirits and by nasal ingestion of tobacco juice and perfumes.

Two hundred seventy-eight different medical conditions were recorded. Most plants were used for the treatment of multiple ailments. The large variety of conditions is grouped into 72 main categories.

#### 5.3.1. Magical Uses

Mental, neurological, and psychosomatic disorders are highly prevalent on a global scale. The burden of mental health problems has been seriously underestimated. Although neurological problems are only responsible for about 1% of global deaths, they contribute to over 11% of the global disease burden. It is estimated that this share will rise to 15% by 2020 [158]. Western medicine often offers little help for patients afflicted by these disorders.

Healing altars (*mesas*) in Northern Peru often follow the old tradition by including a large variety of “power objects,” frequently with a “pagan” background. Objects such as seashells, pre-Columbian ceramics, staffs, and stones. are very common on Peruvian *mesas* and are blended with Christian symbols such as crosses and images of saints. Treatments are most often performed in the homes of the individual healers, who normally have their *mesas* set up in their backyards. Healers also treat patients at altars and consultation chambers (*consultorios*) in their homes, at sacred sites in the countryside, or at sacred lagoons high up in the mountains. A curing ceremony normally involves purification of the patient by orally spraying blessed and enchanted herbal extracts on the whole body to fend off evil spirits and by “Spiritual Flowerings” (*baños de florecimiento*). In most cases, the cleansing of the patients involves drinking boiled *San Pedro* juice and the nasal ingestion of tobacco juice and perfumes. Sometimes extracts of Jimson weed (*Datura ferox*), *Brugmansia *spp., and tobacco are also used to purify the patients. While the incantations used by healers during their curing sessions include Christian components (e.g., the invocation of Christ, the Virgin Mary, and any number of saints), references to Andean cosmology (e.g., to the *apus* or the spirits of the mountains) are very common. The use of guinea pigs as diagnostic instruments is standard in Northern Peru [24, 159–162].

Traditional Medicine is also gaining more attention by national governments and health providers. Peru's National Program in Complementary Medicine and the Pan American Health Organization recently compared Complementary Medicine to allopathic medicine in clinics and hospitals operating within the Peruvian Social Security System [14].

*Mal Aire* (Bad Air), *Mal Viento* (Bad Wnd), *Susto* or *Espanto* (Fright), *Mal Ojo* (Evil Eye), and *Daño *or *Brujería* (Sorcery) are seen as very common illnesses in Andean society. Causes include sudden changes in body temperature (*Mal Aire, Mal Viento*), any kind of shock (*Susto, Espanto*), “humors” or spells cast by other people (*Mal Ojo*), poisoned food, and curses. (*Daño, Brujeria*). Medical problems caused by outside influences were reported in a wide variety of studies [70, 163]. The Western concept of “psychosomatic disorders” comes closest to characterizing these illnesses.

These illness categories are deeply rooted in Andean society, and Western medicine does not offer efficient alternatives to traditional treatment. This might explain why this category has still such an outstanding importance. Treatment in many cases involved the participation of the patient in a cleansing ceremony or *limpia*. This could either be a relatively simple spraying with perfumes and holy water or an allnight ceremony involving the healer's curing altar (*mesa*). In the days after an all-night ceremony, patients are normally treated with a *baño de florecimiento* (flowering bath) in order to relieve them of any remaining adversary symptoms or spirits. In addition, patients frequently receive *seguros* (herbal amulets) for protection against further evil influences and for good luck. *Seguros* are flasks filled with powerful herbs, as well as perfumes, pictures of saints, and the hair and fingernails of the patient.

The enormous number of plant species used for the treatment of psychosomatic disorders indicates that the *curanderos* of Northern Peru are valued specialists who are consulted mainly for these conditions. This is all the more interesting since Western medicine has still not found efficient treatments for psychosomatic disorders. The plant species used for “magical or ritual” disorders come mostly from the high Andes, especially from the vicinity of sacred lakes, since plants from those regions are regarded as especially powerful. This links the present day curing practices directly to ancient Andean cosmology. The use of purgatives and laxatives, and to literally “expel” evil spirits is also very common.

A total of 222 plant species belonging to 172 genera and 78 families were documented and identified as herbal remedies used to treat nervous system problems in Northern Peru. Most species used were Asteraceae (36 species, 16.21%), followed by Solanaceae (15 species, 6.76%) and Lamiaceae (14 species, 6.31%). The most important nervous system families are somewhat overrepresented in comparison to the overall medicinal flora, while some other medicinally important families (e.g., Poaceae, Cucurbitaceae, and Euphorbiaceae) are completely missing or underrepresented from the nervous disorder portfolio [126].

The majority of herbal preparations were prepared from the whole plant (31.56%), while the leaves (24.48%), stems (21.24%), and flowers (8.55%) were used less frequently. Whole plants and stems were more often used than characteristic for the overall medicinal preparations found in the region [126]. This indicates that the local healers count on a very well developed knowledge about the properties of different plant parts. In over 60% of the cases, fresh plant material was used to prepare remedies, which differs slightly from the average herbal preparation mode in Northern Peru. Interestingly, only about 36% of the remedies were applied orally, while the majority was applied topically (46.65%), often as bath, and the remaining ones were used as spiritual safeguard (*seguro*). This is different from the regional average of application and underlines the importance of spiritually oriented treatments. Over 79% of all remedies were prepared as mixtures with multiple ingredients by boiling plant material either in water or in sugarcane spirit.

Little scientific evidence exists to date to prove the efficacy of the species employed as nervous system remedies in Northern Peru. Only 24% of the plants found or related species in the same genus have been studied at all. Apiaceae, however are particularly well documented. López et al. [38] documented that neurophysiological activity in *Ammi majus* seeds. Celery (*Apium graveolens*) is wisely used traditionally and has been found to be neuroactive [164–167]. Activity against anxiety and stress was found in *Coriandrum sativum *[168], *Centella asiatica*, a species closely related to *Hydrocotyle* spp. [169–177], and *Petroselinum* sp. [178]. *Thevetia peruviana*, frequently employed in Peruvian traditional medicine, was found to be neurotoxic [179, 180]. Many members of the sunflower family are known to contain large amounts of Pyrrolizidine alkaloids and are also rich in other interesting compounds. Not surprisingly, Asteraceae are of high medicinal importance. Yarrow (*Achillea millefolium*) showed neurological activity [181]. *Artemisia* spp. are the prime source of Artemisinin, now employed as antimalarial. However, various species were found to be neuroactive and to act as neurotoxicity inhibitors [182–195]. *Baccharis* SERRATIFOLIA showed neuroactivity [196]. The neurological effects of Chamomile (*Matricaria* sp.), in particular its activity as sedative are well studied [174, 197–200]. *Senecio* sp. [201, 202], *Gynoxys *sp. [203], and *Tagetes* sp. [204–206] have also shown antidepressant effects.

One of the most widely used and studied neuroactive plant genera is *Hypericum* sp. (St. Johns Wort). Species of this genus are widely used in Peru, and *in vitro* as well as *in vivo* studies have long shown its efficacy [207–210]. Similarly important species of Lamiaceae include *Melissa officinalis *[211–214], *Lavandula* sp. [209, 214, 215], and *Origanum majorana* [192]. *Ocimum sanctum *has been used in Ayurvedic preparations for millennia, and other species of the genus have shown neurophysiological efficacy as well [216–220]. *Salvia* sp. has been closely studied since SALVATORIN A was found effective in therapy [213, 221–224].

Chinese Skullcap (*Scutellaria baicalensis*) and other species of the genus *Scutellaria *are employed to treat memory loss and psychological disorders [171, 225–227]. Okuyama et al. [228] and D. Singh and A. Singh [180] reported on the neurotoxicity of *Jatropha* sp. and [229] found neuroactive compounds in *Cyperus* sp., *Sida* sp., *Myristica fragrans* [230, 231], *Alchemilla* sp. [232], *Rubus* sp. [233], *Gardenia* sp. [234], *Ruta graveolens* [235], *Passiflora* sp. [212, 236, 237], *Tilia *sp. [212, 237–241], *Iresine* sp. [242, 243], *Ascophyllum* sp. [244], and *Aloysia* sp. [245, 246] all show anxiolytic properties. Many species of clubmoss (*Huperzia* spp.) are used for cleansing baths and as admixtures to hallucinogenic preparations. The bioactivity of their compounds, for example, Huperzine A, has been widely demonstrated [247]. Members of the citrus family (*Citrus *spp) are well-known calmatives [248–253]. *Valeriana *spp. are well known and proven antidepressants and are widely used as mild sedative [174, 243, 244, 254–274]. The genus is used for the same purpose in Northern Peru. [275] reported on the use of *Mikania* sp. Lastly, a multitude of species is used in Northern Peru for their psychoactive properties. Traditionally, coastal as well as Amazonian cultures employed hallucinogenic snuffs, often derived from *Anadenanthera* sp. or* Virola *sp. [276–281]. However, the use of hallucinogenic snuffs has all but disappeared from the region [126, 157].

Many Solanaceae have been used in traditional medicine for millennia and maintain still high ritual importance. However, in many cases, these plants are only used as “plants of last resort,” because the local healers are well aware of their toxicity. *Brugmansia* spp. and *Datura *spp. are sometimes added to mixtures of San Pedro cactus and Tobacco juice and inhaled through the nostrils or are added to cleansing baths. The bioactivity of the alkaloids contained in this species is well documented [46, 282–298]. Plowman [299] reported on the use of *Brunfelsia* sp. as hallucinogens. *Nicotiana tabacum *and* N. rustica *still have wide ceremonial importance in the Native American as well as Andean communities, and both species can have profound psychoactive effects in high dosage [300–305].

The most widely known neuroactive species in South America is probably the San Pedro cactus (*Echinocereus pachanoi*), an ingredient of almost every healing ceremony along the coast between Ecuador and Bolivia, and also widely employed in the highlands. Mescaline, the main active compound, has previously been used in western psychotherapy but was subsequently banned. The effect of San Pedro concoctions or isolated compounds is widely reported [47, 306–315]. Ayahuasca (*Banisteriopsis caapi*) however is more widely used for spiritual experiences, and its central nervous system activity is well documented [316–323].

#### 5.3.2. Respiratory System

The WHO reports that respiratory illnesses are of high importance as a cause of death and morbidity at a global scale. WHO elaborated a Strategy for Prevention and Control of Chronic Respiratory Diseases (CRDs), [324], and respiratory problems are a major cause for infant deaths in Peru [325].

A total of 91 plant species belonging to 82 genera and 48 families were documented and identified as respiratory system herbal remedies in Northern Peru. Most species used were Asteraceae (15 species, 16.67%), followed by Lamiaceae and Fabaceae (8.89% and 5.56%). Most other families contributed only one species each to the pharmacopoeia. The most important families are clearly similarly well represented in comparison to the overall medicinal flora, although some other medicinally important families (e.g., Euphorbiaceae, Lycopodiaceae, and Cucurbitaceae) are completely missing from the respiratory portfolio [125].

The majority of respiratory disorder herbal preparations were prepared from the leaves of plants (27.69%), while the whole plant (18.46%), flowers (13.85%), and stems (17.69%) were used less frequently [125]. This indicates that the local healers count on a very well developed knowledge about the properties of different plant parts. In almost 55% of the cases, fresh plant material was used to prepare remedies, which differs little from the average herbal preparation mode in Northern Peru. About 86% of the remedies were applied orally, while the remaining ones were applied topically. Over half of all remedies were prepared as mixtures of multiple ingredients by boiling plant material either in water or in sugarcane spirit.

Respiratory disorders are so common globally, and over-the counter remedies, both allopathic and complementary, so frequently sold, that much effort has been put into the verification of traditional remedies. Almost 50% of the plants found in the respiratory pharmacopoeia of Northern Peru or their congeners have been studied for their medicinal properties. The original hypothesis that many species employed for respiratory illnesses would be nonnative, introduced to treat diseases that were originally also introduced by colonialists, did not hold; however, Quite contrarily, many remedies for respiratory ailments are native to the study area [125]. From this perspective, it is surprising to see how many species have actually been studied at least preliminarily. Biella et al. [326] report on the activity in an extract of *Alternanthera*. Braga et al. [327] worked on *Schinus molle*. Other examples include *Apium graveolens* [328], *Acmella* [329], *Clibadium* [330], *Eupatorium* [331], *Flaveria* [332], *Perezia* [333], *Senecio* [334], *Tagetes* [335], *Alnus* and *Sambucus* [336], *Jacaranda* [337], *Raphanus* [338], *Cordia* [339], *Scabiosa* [340] *Bursera* [341], *Erythroxylum* [342], *Myroxylon* [343], *Prosopis* [344], *Lavandula* [334, 345], *Cinchona* [288], *Juglans* [346], *Uncaria* [347, 348], *Cymbopogon* and *Cinnamomum* [349, 350], *Plantago* and *Eucalyptus* [351, 352], *Malva* and *Alcea* [353] *Dracaena* [354], *Allium* [355–357], *Rubus* [358, 359], *Stachys* [360], *Satureja* [335, 361], *Salvia* p. [362], and *Thymus* [351].

#### 5.3.3. Urinary System (Kidneys, Bladder)

The recent WHO report on urinary tract infections (UTI) indicates that UTI are one of the most common bacterial infections seen, in particular in children. It has been estimated that UTI are diagnosed in 1% of boys and 3–8% of girls. In the first year of life, UTI is more prevalent in boys with rates of 2.7% compared with 0.7% in girls. The reported rate of recurrent UTI is around 12–30% with risk greater in infants <6 months, severe vesicoureteric reflux, and abnormal nuclear renal scans at time of first infection [363].

Studies have shown a higher UTI prevalence of 8–35% in malnourished children, with the risk of bacteriuria increasing significantly with the severity of malnutrition [363].

A total of 69 plant species belonging to 61 genera and 43 families were documented and identified as herbal remedies for kidney and urinary tract problems in Northern Peru. Most species used were Asteraceae (8 species, 11.43%), followed by Fabaceae and Poaceae (both 5 species, 7.14%). All other families mostly contributed only one species each to the pharmacopoeia. The most important families are represented similarly as in the overall medicinal flora, while some other medicinally important families (e.g., Lycopodiaceae, Cucurbitaceae) are completely missing from the kidney portfolio [126].

The majority of kidney herbal preparations were prepared from the whole plant (27.78%), while the leaves of plants (25.56%), flowers (12.22%) and stems (16.67%) were used less frequently [126]. This indicates that the local healers count on a very well developed knowledge about the properties of different plant parts. In almost 64% of the cases fresh, plant material was used to prepare remedies, which differs little from the average herbal preparation mode in Northern Peru. About 88% of the remedies were applied orally, while the remaining ones were applied topically. Over half of all remedies were prepared as mixtures of multiple ingredients by boiling plant material either in water or in sugarcane spirit.

Kidney and urinary system problems are very common globally, but allopathic treatments, in particular with regard to renal calculi, are mostly focused on dilation of the ureter, and pain management. Although a large number of plants are used in traditional medicine to treat this problem, less than 35% of the plants found in Peru or their congeners have been studied for their medicinal properties. Kim et al. [194] report on the kidney-protective effects of *Brassica* root extract. Efficacy in *Smallanthus sonchifolius* and *Lepidium meyenii*, both neglected Andean crops, and the latter very frequently sold in the herbal supplement industry [126]. Other medicinals with positive effects on the urinary system that were at least exposed to some preliminary research were *Aloe* [364], *Annona* and *Citrus* [365], *Dioscorea* and *Hydrocotyle* [366, 367], and *Plantago* [368]. Lans [369] published a long list of remedies for kidney problems from research in Trinidad and Tobago. *Arctium lappa* [370], *Zea mays* [371], many species of *Equisetum* [371, 372], and especially species of *Phyllanthus* and *Tribulus* [373, 374] have shown efficacy in urolithiasis. The main problem from a patient perspective lies however in the fact that many species, for example, of *Phyllanthus*, are highly similar, while only a few display the desired effect.

Kidney and urinary tract diseases are a major health challenge worldwide. Many plant species are traditionally used for kidney disease treatment, and some have been investigated for their efficacy with positive results. An often-limiting factor to these investigations is lack of comprehensive ethnobotanical data to help choose plant candidates for potency/efficacy tests. Since the plant parts utilized in preparation of kidney remedies are reported in this survey, it serves as an indication of species that may need further ecological assessment on their regeneration status.

#### 5.3.4. Rheumatic Problems

The National Institutes of Health (NIH) reports that an estimated 23.5 million Americans suffer from autoimmune diseases and that this number is expected to grow. Medical research has currently identified 80–100 autoimmune diseases, and 40 additional diseases are suspected to have an autoimmune basis. Autoimmune diseases collectively rank in the top ten leading causes of death for women aged from adolescents up to age 64. In Western medicine, the most common treatments are immunosuppressants, which are known to have devastating long-term side effects [375].

The housing conditions already described, as well as difficult working conditions, lead to a wide spectrum of muscular-skeletal disorders, including rheumatism, arthritis, and bone and muscle pain. A total of 55 plant species belonging to 53 genera and 43 families were documented and identified as autoimmune herbal remedies in Northern Peru. Most species used were Fabaceae (4 species, 7.27%), followed by Rosaceae and Myrtaceae (both 3 species, 5.45%). All other families contributed only one or two species each. The most important families are clearly overrepresented in comparison to the overall medicinal flora, while some other medicinally important families (e.g., Asteraceae, Lamiaceae, Euphorbiaceae, Apiaceae, Lycopodiaceae, and Cucurbitaceae) are less commonly used for the treatment of autoimmune disorders and pain or are completely missing from the portfolio [126].

The majority of the herbal preparations were prepared from the leaves of plants (35%), while the whole plant (21.25%) and stems (17.5%) were used less frequently [126]. This indicates that the local healers count on a very well-developed knowledge about the properties of different plant parts. In 60% of the cases fresh plant material was used to prepare remedies, which differs little from the average herbal preparation mode in Northern Peru. Only about 55% of the remedies were applied orally, while the remaining ones were applied topically. This is little different from the regional average of application. Over half of all remedies were prepared as mixtures of multiple ingredients by boiling plant material either in water or in sugarcane spirit.

Very little western scientific evidence exists to prove the efficacy of the species employed as remedies in Northern Peru to treat autoimmune problems. Less than a pitiful 22% of the plants found or their congeners have been studied at all for their medicinal properties. Garlic (*Allium sativum*) is probably the most widely studied immunomodulating plants, and scientific evidence for its efficacy is quite common [353, 376–381]. Likewise, the widely marketed cat's claw (*Uncaria guianensis*), widely overharvested, and often falsified [126] has been studied intensively [382–385], and the simple stinging nettle (*Urtica dioica*) long used as anti-inflammatory in many traditional medicine systems has been proven to show efficacy [384, 386–392]. In the Middle East, Ratheesh et al. [393, 394] successfully showed activity in (*Ruta graveolens*). However, these studies are rare examples of in-depth assessments of a few well-known species. Few other plants have seen much research on their immunoregulating activity. *Alternanthera tenella* [326, 395], *Baccharis *spp. [396], *Spartium junceum* [397], *Pinus* sp. [398, 399], and *Plantago* sp. [351, 400] are some few exceptions. This is the more surprising as arthroid diseases are very common, and hardly any study has been attempted to cover the properties of a wider range of species as alternative to allopathic for treatment [401–404].

#### 5.3.5. Internal Organs (Liver, Gallbladder)

Disorders of internal organs fall far behind the most commonly treated medical conditions [126]. This is an indication that *curanderos* in Northern Peru are to a large extent specializing in the treatment of psychosomatic disorders and that “bodily” illnesses are treated more as a sideline. However, a large number of plant species were used by local healers to treat liver and gallbladder ailments.

A total of 51 plant species belonging to 43 genera and 31 families were documented and identified as liver and gallbladder herbal remedies in Northern Peru. Most species used were Asteraceae (9 species, 17.66%), followed by Euphorbiaceae (4 species, 7.85%) and Gentianaceae (3 species, 5.89%). All other families contributed only one or two species each to the pharmacopoeia. Asteraceae are clearly over-represented in comparison to the overall medicinal flora, while some other medicinally important families (e.g., Solanaceae, Lycopodiaceae, Cucurbitaceae, and Rosaceae) are completely missing from the liver ailment portfolio [126].

The majority of herbal preparations employed for liver ailments were prepared from the whole plants (35.38%), while the leaves (24.61%), flowers (9.23%), and stems (12.32%) were used less frequently. Whole plants were more often used than characteristic for the overall medicinal preparations found in the region, while stems of plants were employed much less frequently [126]. This indicates that the local healers have a less well-developed knowledge about the constituents of individual plant parts in the case of liver and gallbladder treatments than for other applications [126]. In almost 65% of the cases, fresh plant material was used to prepare remedies, which differs little from the average herbal preparation mode in Northern Peru. Most of the remedies were applied orally (over 90%), while the remaining ones were applied topically. This is highly different from the regional average of application. Over 71% of all remedies were prepared as mixtures with multiple ingredients by boiling plant material either in water or in sugarcane spirit. This indicates that the local healers have a very profound knowledge about the synergistic effects of plants in multi-ingredient preparations.

Almost no scientific evidence exists to date to prove the efficacy of the species employed as liver and gallbladder remedies in Northern Peru. Only 8% of the plants found or related species in the same genus have been studied at all. *Spartium junceum*, *Malva* spp., and *Plantago* spp. are used in liver ailments [405], but no other species found in Northern Peru have been shown to be effective against these conditions.

#### 5.3.6. Diarrhea, Stomach Problems, and Other Intestinal Ailments

Foodborne diseases are a serious public health problem worldwide. Some foodborne diseases are well recognized but have recently become more common. Outbreaks of salmonellosis have been reported for decades, but, within the past 25 years, the disease has increased in incidence on many continents. While cholera has devastated much of Asia and Africa for years, its introduction for the first time in almost a century on the South American continent in 1991 makes it another example of an infectious disease that is both well recognized and emerging. While cholera is often waterborne, many foods also transmit infection. Infection with *Escherichia coli *serotype O157:H7 (*E. coli*) was first described in 1982. Subsequently, it has emerged rapidly as a major cause of bloody diarrhea and acute renal failure. Outbreaks of infection, generally associated with beef, have been reported in Australia, Canada, Japan, United States, in various European countries, and in southern Africa [406].

A total of 75 plant species belonging to 62 genera and 39 families were documented and identified as herbal remedies for intestinal ailments in Northern Peru. Most species used were Lamiaceae (13.33%), followed by Asteraceae and Rutaceae (both 5 species, 6.67%). Most other families contributed only one species each to the pharmacopoeia. The most important anti-infectious families are clearly over-represented in comparison to the overall medicinal flora, while some other medicinally important families (e.g., Asteraceae) are much less important [126].

The majority of anti-infectious herbal preparations were prepared from the leaves of plants (29.25%), the whole plant (22.64%), and stems (16.04%). This indicates that the local healers count on a very well developed knowledge about the properties of different plant parts. In almost 60% of the cases fresh plant material was used to prepare remedies, which differs little from the average herbal preparation mode in Northern Peru. Interestingly, only about 83% of the remedies were applied orally, while the remaining ones were applied topically. Over half of all remedies were prepared as mixtures of multiple ingredients by boiling plant material either in water or in sugarcane spirit.

Large parts of the species used for intestinal disorders in Northern Peru are introductions from other parts of the world, especially Europe. Many of these are well known, and almost 50% of the plants found in this study have shown efficacy in scientific studies.

A large number of Apiaceae are used for their stomach calming and antibacterial effects (e.g., *Apium graveolens* [407, 408]; *Foeniculum vulgare* [409]; and *Pimpinella anisum* [410–412]. Coconut (*Cocos nucifera*) showed antiulcerogenic activity [413], as did Yarrow (*Achillea millefolium*) [414, 415], as well as *Arctium lappa *[416–418]. Schütz et al. [419], H.-G. Grigoleit and P. Grigoleit [420], and You et al. [421] reported that *Taraxacum officinale* (Dandelion) relieved oxidative stress and has gastroprotective effects and *Capsella bursa-pastoris* is well known for its antiinflammatory and hepatoprotective function [422–424]. Well-known medicinal plants, for example, *Hypericum* sp. [425], *Croton lechleri* [426, 427], and *Desmodium gangeticum* [428], also have antiulcer activity. *Hyptis pectinata* showed hepatoprotective activity [429].

Lamiaceae were particularly effective against gastrointestinal problems. *Mentha piperita* showed antibacterial and calmative effects [430], while *Origanum vulgare* and* Origanum majorana* had pronounced antihyperlipidemic and antioxidant effects [431]. Rosemary (*Rosmarinus officinalis*) has potential to relieve oxidative stress and is strongly antibacterial [432–435]. Kawagishi et al. [436] found strong liver-protective activity in Avocado (*Persea americana*), and Khasina et al. [437] reported gastro-protective effects of Duckweed (*Lemna minor*). A variety of Lythraceae is also well known for their antioxidant and antibacterial properties, as studies in the Americas [433, 438], and the Near- and Middle East [438–440] indicate.

Maity et al. [441], Yadav and Bhatnagar [442], and Chaturvedi et al. [443] demonstrated the efficacy of Indian spices as gastroprotective agents. *Passiflora* sp. as well as *Piper *sp. and rice (*Oryza *sativa) were found to demonstrate strong anti-bacterial and antioxidant properties [411, 444–448]. Only recently antiinflammatory activity of *Citrus* sp., and *Ruta graveolens *[449] was demonstrated and even plants that have long been used in codified traditional medicine for their gastro-protective function has only been studied in detail during the last few years, for example, pomegranate (*Punica granatum*) [450–454] and green tea (*Camellia sinensis*) [455, 456].

#### 5.3.7. Reproductive Problems and Female Health

According to 1999 WHO estimates reproductive problems, including, 340 million new cases of curable sexually transmitted diseases (STIs; syphilis, gonorrhoea, chlamydia and trichomoniasis) occur annually throughout the world in adults aged 15–49 years. In developing countries, STIs and their complications rank in the top five disease categories for which adults seek health care. Infection with STIs can lead to acute symptoms, chronic infection and serious delayed consequences such as infertility, ectopic pregnancy, cervical cancer, and the untimely death of infants and adults [457].

A total of 105 plant species belonging to 91 genera and 62 families were documented and identified as herbal remedies for reproductive problems in Northern Peru. Most species used were Asteraceae (9.52%), followed by Lamiaceae and Fabaceae (8.57% and 6.67%). Other families were less important, and 44 contributed only one species each to the pharmacopoeia. The most important families are clearly represented very similarly to their overall importance in the local pharmacopoeia [126].

The majority of herbal preparations for reproductive issues were prepared from the leaves of plants (22.72%), the whole plant (21.97%), and stems (21.21%), while other plant parts were used much less frequently. This indicates that the local healers count on a very well developed knowledge about the properties of different plant parts. In almost 62% of the cases, fresh plant material was used to prepare remedies, which differs little from the average herbal preparation mode in Northern Peru. Over 70% of the remedies were applied orally, while the remaining ones were applied topically. Many remedies were prepared as mixtures of multiple ingredients by boiling plant material either in water or in sugarcane spirit.

Little scientific evidence exists to prove the efficacy of the species employed as reproductive disorder remedies in Northern Peru. Only 34% of the plants found or their congeners have been studied at all for their medicinal properties. *Aloe* spp. are known to have oestrogenic activity [369, 458]. Adams and Garcia [459] reported that *Artemisia* spp. had effects on female health amongst the Chumash. A variety of other Asteraceae have been shown to be used against menopausal symptoms (*Clibadium* [75]; *Matricaria* [362, 460, 461]; *Taraxacum* [462, 463]). Lans [369] found hormonal effects in *Cordia* sp., while [463–467] reported on anti-fertility effects of *Dioscorea *sp. *Cupressus* sp. are well known abortifacients [468], while pumpkin seed oil showed testosterone-inhibitory effects [369, 469–471]. *Chamaesyce* sp. showed promise in the treatment of male infertility, while *Mimosa* sp. on the contrary are used to reduce spermal fertility [369, 472].

A wide range of Lamiaceae have been shown to exhibit contraceptive efficacy, and the same species are used in Peru for similar purposes (*Mentha* spp. [473–476]; *Ocimum* spp. [477–480]; *Origanum majorana* [476, 481, 482]; *Rosmarinus officinalis* [472]). Similar efficacy has been shown for *Sanguisorba officinalis *[483], and *Ruta graveolens* [369, 484–487].

Various species of *Passiflora* have aphrodisiac activity [488–491], and *Myristica fragrans* as well as *Syzygium aromaticum* [492], and extracts of *Lantana camara* [493, 494] and *Pilea* spp. [369] fulfil the same purpose, while *Portulaca oleracea* showed efficacy in relieving uterine bleeding [495, 496].

#### 5.3.8. Heart and Circulatory System

Cardiovascular diseases are collectively the number one cause of death on the globe, accounting for over 30% of all deaths worldwide, 80% of which occur in lower income countries with often little western healthcare available. Lower income groups have generally a higher prevalence of risk factors [158]. Traditional Medicine is used globally and has rapidly growing economic importance. In developing countries.

Traditional healers are frequently consulted to treat heart problems and disorders of the circulatory system. The healers encountered used a wide variety of terms relating to heart problems, that in part generalized the condition (e.g., “heart disease”), included references to conditions as underlying cause of heart problems (e.g., “cholesterol”), or simply used terms to indicated treatment options (e.g., “blood irrigation” as term referring to “thin” a patients blood, “blood purification,” or “refreshing the heart” as terms indicating a process cleansing the blood from suspected toxins, or “blood circulation,” indicating a treatment that would improve circulation). The use of western style biomedicinal terms is not surprising, given that all informants were of Mestizo origin and lived in an urban environment.

Most treatments of the circulatory system involved the purification of the blood in order to improve the general condition of the patient. In addition, the fashionable concept of “weight management” and conditions related to obesity has entered into the domain of Peruvian healers. All healers readily acknowledge the negative influence of high cholesterol levels, and plant remedies were used specifically to lower cholesterol as well as weight loss therapies, while plants used for weight gain were insignificant.

A total of 60 plant species belonging to 52 genera and 33 families were documented and identified as heart herbal remedies in Northern Peru. Most species used were Asteraceae (7 species, 11.67%), followed by Lamiaceae (6 species, 10%), and Solanaceae (4 species, 6.67%). Fabaceae, Amaranthaceae, and Cucurbitaceae each contributed 3 species (5%) to the heart pharmacopoeia. All other twenty-seven families contributed only one or two species each to the pharmacopoeia. Asteraceae are in general under-represented as heart remedies in comparison to the medicinal flora used in Northern Peru; Lamiaceae and Euphorbiaceae are clearly over-represented in comparison to the overall medicinal flora, while some other medicinally important families (e.g., Poaceae, Lycopodiaceae, and Rosaceae) are completely missing from the heart portfolio [126].

The majority of heart remedies were prepared from whole plants (37.18%), while the leaves (24.36%), stems (15.38%), and flowers (7.69%) were used less frequently. Whole plants were more often used than characteristic for the overall medicinal preparations found in the region [126]. In almost 70% of the cases, fresh plant material was used to prepare remedies, which differs little from the main herbal preparation mode in Northern Peru. Over 90% of the remedies were applied orally, while the remaining ones were applied topically. This is very different from the regional average of application. Over 65% of all remedies were prepared as mixtures with multiple ingredients by boiling plant material either in water or in sugarcane spirit. This indicates that the local healers have a very profound knowledge about the synergistic effects of plants in multi-ingredient preparations.

Little scientific evidence exists to date to prove the efficacy of the species employed as heart remedies in Northern Peru. Only 33% of the plants found or related species in the same genus have been studied at all. *Ambrosia* sp. shows some promise in the treatment of myocardial infarction [497]. *Citrullus* spp., *Sanguisorba *sp., *Viola* sp., *Lavandula* sp., and *Smilax* spp. are used in the Middle East to treat heart problems [209] the latter species are with good indications for clinical efficacy [498]. *Cucurbita* spp. and *Cuphea* spp. were found that to be effective in Brazil [499, 500]. The use and efficacy of *Lathyrus* sp., is widely documented [501–505]. Lev [506] found *Tamarindus* sp., *Ocimum *spp., *Viola *sp. and* Rosmarinus officinalis* are used for heart conditions in Israel. Plantain (*Plantago* spp.) has well documented cardiac effects [507, 508], as do various species of *Citrus* spp. [398, 509, 510], while *Peperomia* spp. and *Passiflora* spp. are often employed as folk remedies in the Caribbean [369].

#### 5.3.9. Inflammation and Bacterial Infections

Bacterial infections and inflammation are among the ailments responsible for a large number of deaths worldwide and are often treated by traditional healers [125, 511].

A total of 96 plant species belonging to 84 genera and 46 families were documented and identified as anti-infective herbal remedies in Northern Peru. Twenty percent of the species were introductions, while 80% belonged to the native flora of Peru. Most species used belong to Asteraceae (18.95%), followed by Fabaceae and Euphorbiaceae (7.37% and 5.26%). Most other families contributed only one species each to the pharmacopoeia. The most important anti-infectious families were over-represented in comparison to the overall medicinal flora, while some other medicinally important families (e.g., Lycopodiaceae, Cucurbitaceae) are completely missing from the anti-infective portfolio.

The majority of herbal preparations were prepared from the leaves of plants (31.34%), while the whole plant (18.66%), flowers (12.69%), and stems (17.16%) were used less frequently. In almost 67% of the cases, fresh plant material was used to prepare remedies. Only about 55% of the remedies were applied orally, while the remaining ones were applied topically. Over half of all remedies were prepared as mixtures of multiple ingredients by boiling plant material either in water or in sugarcane spirit.

Infections, in particular by strains of *Staphylococcus aureus,* are very common, and increasingly difficult to treat, due to widespread formation of drug resistance. Fungal infections, due to the structure of the organisms involved, have always been a hard task to treat. Given the high importance of infections, it is not surprising that anti-infective agents are high on the list for drug development, and a large number of species used traditionally have undergone screening. Almost 43% of the plants used in Northern Peru to treat infections or their congeners have been studied for their medicinal properties, and the respective references are given in the following section. Biella et al. [326] reported on the antibacterial efficacy of *Alternanthera tenella*. Mango (*Mangifera indica*) has shown antibacterial efficacy in a wide variety of studies [512–515]. Compounds of *Schinus molle* showed anti-inflammatory activity [516]. Oleandrin, isolated from *Nerium oleander*, was found to be active in inhibiting the kappa-B inflammation cascade [517]. Rinaldi et al. [518] showed anti-inflammatory activity in *Cocos nucifera*. Chinese traditional preparations like Guizhi-Fuling, containing *Cinnamomum vulgare*, have shown anti-inflammatory activity also [519–522]. A wide range of Asteraceae have strong anti-bacterial and anti-inflammatory properties. Benedek and Kopp [523] and Nemeth and Bernath [524] found anti-infective potential in Yarrow (*Achillea millefolium*). Many species of *Baccharis* proved effective [525, 526], as did *Bidens pilosa* [527–529]. Other efficacious members of the sunflower family include *Eupatorium* [530–534], *Matricaria recutita* [535], *Tagetes patula* [536], and *Taraxacum officinale* [430, 537]. *Capsella bursa-pastoris* was found to act as anti-inflammatory [422], while *Dioscorea* was found to have immunostimulating properties [538, 539]. Zhang et al. [540] reported pain-relieving properties in *Gaultheria yunnanensis*. Jones [427] found antibacterial activity in *Croton lechleri* (Sangre de drago). Other examples for plants with anti-bacterial potential found in Peru include *Manihot esculenta* [541], *Solanum nigrum* and *Ricinus communis* [542, 543], *Solanum* sp. [544], *Caesalpinia* spp. [455, 545], *Mezoneuron benthamianum* [546], *Desmodium triflorum *[547], *Leucaena leucocephala* [548], and Red clover (*Trifolium pratense*) [549]. Salvinorin A, extracted from *Salvia* spp. [550–552] showed immunomodulatory properties. Other Lamiaceae with antiinfective compounds include *Satureja hortensis* [553]. *Buddleja* spp. were found to be mainly antiinflammatory and antioxidant [554, 555]. *Plantago* sp. [556], *Cynodon dactylon* [557], *Polypodium* sp. [558, 559] and *Uncaria* sp. [560], all commonly used in Peru, show cox-2 inhibition, and thus anti-inflammatory properties. Cat's claw (*Uncaria tomentosa*) has long been marketed as traditional anticancer remedy, leading to serious over-harvesting and flooding of the market with adulterated material [126]. Sandoval-Chacón et al. [560], Mur et al. [383], and Hardin [385] could confirm antiinflammatory properties of the species. Calvo [561] and Speroni et al. [562] confirmed analgesic activity in *Verbena* sp. A few plant groups have been studied more in depth. Rutaceae (*Citrus* spp.) have proven antiinflammatory effect [563–567], as did *Gardenia* sp. [568, 569], while many species of *Smilax* exhibit immunomodulatory effects [402, 570–572]. Vargas et al. [573] found antiinflammatory properties in *Passiflora alata* and *Passiflora edulis.*

#### 5.3.10. Malaria and Fever

Malaria is still a major global public health problem in most tropical countries. It is thought that malaria is by far the most serious tropical disease causing one to two million deaths per year, and it plays a major role in the high mortality seen in infants and children [574, 575]. It is also responsible for miscarriages, premature deliveries, growth retardation, low birth weight, and anemia [576–579].

The World Health Organization (WHO) has estimated that about 2 billion people in over 100 countries are exposed to malaria, with 247 million cases in 2006 alone, and half of the world's population is potentially exposed to the disease [511]. The worsening global economic situation makes it difficult to expand modern health services; hence, effective low-cost delivery medical system is urgently needed [574].

This is even more pressing because the use and misuse of over the counter antimalaria remedies like chloroquine to prevent and treat *falciparum* malaria have led to widespread appearance of resistant parasites [575]. This is complicated by the fact that global warming may lead to expansion of areas in which the ambient temperature and climatic conditions are suitable for *Plasmodium* transmission. Climatic variability has been associated with some of the recent epidemics [578].

A total of 17 plant species belonging to 17 genera and 13 families were documented and identified as anti-malarial herbal remedies in Northern Peru. Most species used were Asteraceae (3 species, 17.66%), followed by Fabaceae and Solanaceae (both 2 species, 11.77%). All other families contributed only one species each to the pharmacopoeia. The most important antimalarial families are clearly over-represented in comparison to the overall medicinal flora, while some other medicinally important families (e.g., Lamiaceae, Euphorbiaceae, Poaceae, and Apiaceae) are completely missing from the antimalarial portfolio [126]. In the context of the questionnaires, healers and venders often referred to “Fever” when talking about malaria. Fever however included a variety of conditions, from fevers accompanying flu to fever as a result of malaria. Malaria was recognized as a parasitic infection, and treated accordingly, while other plant species were used to treat fever as a symptom, mainly focusing on lowering body temperature.

The majority of anti-malarial herbal preparations were prepared from the leaves of plants (38.46%), while the whole plant (26.92%), flowers (15.38%), and stems (11.54%) were used less frequently. Leaves and stems were used more often for malaria treatments than would have been expected in comparison to the overall medicinal preparations found in the region, while seeds of plants were employed much less frequently and other plant parts not at all [126]. This indicates that the local healers count on a very well developed knowledge about the properties of different plant parts. In almost 70% of the cases, fresh plant material was used to prepare remedies, which differs little from the average herbal preparation mode in Northern Peru. Interestingly, only about 55% of the remedies were applied orally, while the remaining ones were applied topically. This is little different from the regional average of application. Over half of all remedies were prepared as mixtures of multiple ingredients by boiling plant material either in water or in sugarcane spirit.

The very limited number of plants employed at the Peruvian coast to treat malaria and fevers might on a first glance surprise, if compared to studies from other regions of the country [580, 581]. However, malaria has always been of relatively minor importance in the coastal desert areas, and thus it is not surprising that few remedies are employed. There are indications that health practices are in the process of changing, and traditional healers start to treat a patient with prepared western remedies (e.g., Aspirin, Primaquine, Malaraquin, or Lariam), although plant preparations are still important [125, 129, 130].

Little scientific evidence exists to prove the efficacy of the species employed as malaria remedies in Northern Peru. Only 41% of the plants found or their congeners have been studied at all for their medicinal properties. *Sambucus* spp. are known to be used against malaria in Trinidad [582], and Stowers et al. [583] showed antiplasmodial activity in an extract of a species of the genus. *Hypericum* spp. are traditionally used in Southern Peru to treat malaria [584], while various species of *Ipomoea* are used in Africa [585–588] and the Philippines [589]. The genus *Salix* is well known as a source of Acetylsalicylic acid, widely used as analgesic and antipyretic. A wide variety of Solanaceae, including species of the genera *Cestrum* and *Solanum*, are widely used as mosquito repellents or as larvicidels [473, 590], or are traditionally used as malaria treatment [582, 586, 587, 591–595], while *Verbena* sp. is known as anti-malarial from Ethiopia [594].

#### 5.3.11. Cancer and Tumors

Forty-seven plant species belonging to 42 genera and 30 families were used by curanderos in Northern Peru to treat cancerous conditions and diabetes symptoms. Most species used were Asteraceae (9 species, 19.15%), followed by Gentianaceae (3 species, 6.37%), and 7 families with 2 species each (4.25%). All other families contributed only one species each to the pharmacopoeia. Asteraceae as the most important anticancer and antidiabetic family is clearly over-represented in comparison to the overall medicinal flora, while most other medicinally important families are either under-represented or completely missing from the portfolio [126].

The majority of anti-cancer and anti-diabetic herbal preparations were prepared from the leaves of plants (30.77%), while the whole plant (20%), stems (20%), and flowers (6.15%) were used less frequently. Leaves and stems were more often used than characteristic for the overall medicinal preparations found in the region, while whole plants were employed less frequently [126]. This indicates that the local healers count on a very well developed knowledge about the properties of different plant parts. In almost 60% of the cases fresh plant material was used to prepare remedies, which differs little from the average herbal preparation mode in Northern Peru. Over 90% of the remedies were applied orally, while the remaining ones were applied topically. This is significantly different from the regional average of application. More than 50% of the remedies included multiple plants.

Little scientific evidence exists to date to prove the efficacy of the species employed as anti-cancer and anti-diabetic remedies in Northern Peru. Only 38.71% of the plants found as diabetes treatments and 17.65% employed as anti-cancer remedies or related species in the same genus have been studied at all. *Schinus molle* is well known for the treatment of diabetes in Bolivia [596] and showed promise against cancer in Brazil [597]. *Thevetia peruviana *and *Arctium lappa* [598] both showed promise in *in vitro* cancer studies.

A wider variety of plants are used as diabetes treatments. *Musa* sp. and* Bidens* spp. are used for this purpose in the Caribbean and Peru [584] and banana is also used in the Middle East [209]. Mulberries (*Morus *sp.) have been found as diabetes remedy both in the Mediterranean [209]. *Mimosa *sp. is a traditional diabetes remedy in India [593], and the same author also reported on *Annona* sp. and *Aloe barbadensis *used for this purpose. *Aloe* has indeed shown some efficacy for diabetes treatment [599, 600]. Johnson [601] found that the Giksan used *Achillea* sp. against diabetes. Bletter [584] reported Cat's claw (*Uncaria tomentosa*) as diabetes plant for the Ashaninka in Peru. *Rubus* sp. is used as anti-diabetic in Nepal [602], and *Ficus *spp., *Smilax* spp., and *Olea europaea* have long been known as diabetes remedies in the Mediterranean and India [209, 603]. Olive has indeed shown to regulate glucose levels [604]. Other studies refer to Artichokes (*Cynara cardunculus*,) [605], Chickpeas (*Cicer arietinum*) [606], *Ocimum* sp. [607, 608], *Citrus *spp. [609], *Phyllanthus *spp. [610], *Ficus* spp. [611], Ginger and Banana (*Zingiber officinale* and *Musa *x* paradisiaca*) [612, 613], Walnut (*Juglans regia*) [209, 604], and *Cestrum* sp. [614].

### 5.4. Parts of Medicinal Plants Used and Mode of Application

Northern Peruvian *curanderos* prefer to use either the leaves (in 25% of all uses) or the whole plant (24%) for the preparation of their remedies. In 19% of the cases, the stems of the plants were used, most commonly together with the leaves. Flowers (10%), seeds (7%), fruits and roots (4% each), bark (3%), fruit peel (2%), and latex and wood (1% each) were only used for a small number of preparations.

Almost two-thirds (64%) of the remedies employed in Northern Peru are prepared using fresh plant material. Many of the introduced species are cultivated in fields and gardens, but the majority of the indigenous species are collected wild. This indicates that a widespread system of plant collectors is needed to supply the fresh plant material needed in Traditional Medicine. Most healers agreed, however, that in most cases dried material could be used if fresh plants were not available. In 36% of all cases, the remedies were prepared using specifically dried plant material. Fresh material was not used in these situations.

Healers in Northern Peru often employ very sophisticated mixtures of a variety of plants in their treatments. The use of single species for treatments was rare. Most commonly, plant material was boiled in water, or in some cases in sugarcane alcohol (*aguardiente*) to extract the active compounds. In some cases, plant material was macerated in cane alcohol or wine for longer periods of time before use.

The *curanderos* all had strikingly exact recipes for treatment, with very specific quantities of plant material used to prepare remedies. These quantities did not differ greatly from one healer to another. Also, the amount of a specific remedy that was given to a patient was very similar among the different *curanderos*.

The most frequent way to administer remedies was to prepare a decoction and ingest it orally (52% of all uses) followed by application as a poultice (38%, plant crushed and/or boiled, and applied). Seven percent of all plant uses entailed the preparation of a *seguro*, a bottle or small flask filled with plant material along with various perfumes. This amulet has to be carried by the patient at all times, or it is placed in the house and used for periodic blessings. *Seguros* contained anything from a handful to more than three-dozen different ingredients. In two percent of the plant uses, the material was employed to fabricate charms, and, in one percent of all applications, the plant material was burned as incense, with the smoke inhaled for treatment.

Many traditional healers rely on herbal preparations, often consisting of complex ingredients and with very specific preparations, to treat their patients' illnesses, rather than just employing single plant extracts. However, studies documenting these preparations and analyzing the composition of the mixtures are almost nonexistent. Most ethnobotanical studies to date document the “use” of single species, without asking the important question if the plants in question are really employed alone, or if they are in fact part of a more complex preparation. Cano and Volpato [615] and Mur et al. [383] were amongst the first authors to respond to this challenge, and reported on plant mixtures employed in Cuba and the Middle East, and Vandebroek et al. [616] demonstrated the great complexity of plant preparations in the Dominican Republic. No information however was available for the very species rich Andean pharmacopoeia.

The present publication attempts to give a detailed overview on the herbal mixtures employed by traditional practitioners in Northern Peru and the specific applications they are used for, in order to provide a baseline for more in-depth studies on efficacy and safety of these preparations, as well as the possible applications in the public health system.

The investigation of plant mixtures used in traditional medicine in Northern Peru yielded a total of 974 herbal preparations used to treat 164 different afflictions [127]. The classification of diseases followed the curandero's terminology. To allow a better overview, the different disease concepts were grouped in more inclusive disease categories, according to their similarity. Psychosomatic disorders were the most outstanding afflictions treated with traditional herbal mixtures, with almost 30% of all recipes applied, followed by respiratory illnesses, female issues, kidney problems, and heart problems. *Susto* (fright), problems of the nervous system, general systemic inflammation, and bronchitis together accounted for almost 25% of all remedies used. In many cases, healers used only one or two common mixtures to treat an illness. This degree of consensus between different healers shows great sophistication in the diagnosis and treatment of specific disorders. On the contrary, when it came to the treatment of unspecific disease categories like “inflammation” or “bronchitis,” every healer seemed to use her/his own specific mixture to treat the problem. This was particularly obvious in the treatment of neurological and psychosomatic problems, for which the majority of plants and mixtures were employed. Up to 49 different preparations were used to treat the same disease. This seems to indicate a high degree of experimentation that is still ongoing in order to find a working cure for unspecific symptoms, and that there is very little consent amongst the individual healers as which cure to employ. This low consensus, especially where spiritual and nervous system/psychosomatic aspects are involved, might also indicate that the individual healers are reluctant to exchange knowledge about their dedicated, specific, and guarded treatment methodology in these areas, while the knowledge about “simple” treatments is much more widespread.

Altogether, 330 plant species, representing almost 65% of the medicinal flora used in the region [127], were applied in mixtures. Of these, 64 species (19.39%) were introductions, which falls within the range of introduced species as percentage of the whole medicinally used flora. Amongst the plants employed, Asteraceae expectedly stood out, and the number of species of this family used was comparable to the percentage of Asteraceae in the medicinal flora of the region [126]. The overwhelming number of plant mixtures contained 2–7 different plant species, although, in the most extreme case, 27 distinct species were included. A large number of species appeared in various mixtures. The plant species for each mixture are listed in the order given by the *curanderos* in order to express the importance of the individual species, rather than providing an alphabetical listing. For a detailed overview on quantities and parts of each plant use, see [126].

The cluster analysis confirmed that mixtures used for applications like inflammations, infections, and blood purification, as well as cough, cold, bronchitis or other respiratory disorders, or urinary infection and kidney problems had similar floristic compositions. However, a few interesting clusters stood out Mixtures used for nervous system disorders, anxiety, and heart problems often had a similar composition, for example, as did mixtures for prostate and bladder problems; kidney problems, gallbladder disorders, diabetes, and cholesterol were treated with the same preparations as were rheumatic illnesses and asthma. Our research suggests that this indicates that the local healers have a very detailed understanding of disease concepts and are choosing their remedies very carefully based on what underlying cause they diagnose; that is, heart problems get treated differently if they are caused by stress, versus a physical agent. Kidney infections are treated differently from kidney problems linked to diabetes and/or obesity.

The floristic composition as well as the complex phytochemistry of traditional herbal mixtures remains woefully understudied. This is the more surprising as traditional one-plant one single-compound based drug discovery efforts have yielded very little results in the last decades and might in fact be an explanation as to why so many plant species that have been documented for a certain use are “inefficient” or “toxic” when introduced to clinical trials.

Our research indicates that a large number of plants used in traditional healing in Northern Peru are employed in often-sophisticated mixtures, rather than as individual plants. Peruvian *curanderos* appear to employ very specific guidelines in the preparation of these cocktails and seem to have a clear understanding of disease concepts when they diagnose a patient, which in turn leads them to often apply specific mixtures for specific conditions. There seems to be a widespread exchange of knowledge about mixtures for treatment of bodily diseases, while mixtures for spiritual, nervous system, and psychosomatic disorders appear to be more closely guarded by the individual healers.

Traditional herbal mixtures, with their wealth of compound fragments and new compounds originating in the preparation process, could well yield new clues to the treatment of a wide variety of disease. The present paper provides detailed baseline information on composition and use of traditional mixtures in Northern Peru, and further studies to compare the compound composition of these preparations versus single plant extracts, as well as investigations comparing efficacy and toxicity of herbal preparations versus their single plant ingredients, are in progress.

### 5.5. Does Traditional Medicine Work? A Look at Antibacterials Used in Northern Peru

Plants with potential medicinal activity have recently come to the attention of Western scientists, and studies have reported that some are bioactive [617]. Potentially active compounds have been isolated from a few of the plants tested [618–622].

In order to evaluate the antibacterial activity of species used in TM in Northern Peru, 525 plant samples of at least 405 species were tested in simple agar-bioassays for antibacterial activity against *Staphylococcus aureus, Escherichia coli, Salmonella enterica typhi*, and *Pseudomonas aeruginosa*. A much larger number of ethanolic plant extracts showed any antibacterial activity compared to water extracts for all antibacterial activity. One-hundred ninety-three ethanolic extracts and 31 water extracts were active against *S. aureus*. In twenty-one cases, only the water extract showed activity (for all bacterial species) compared to ethanol only. None of the aqueous extracts were active against the other three bacteria, with the activity of the ethanolic extracts also much reduced, as only 36 showed any activity against *E. coli* and 3 each against *S. enterica typhi *and *P. aeruginosa. *Eighteen ethanol extracts were effective against both *E. coli* and* S. aureus*, while in two cases, the ethanol extract showed activity against *E. coli *and the water extract showed activity against *S. aureus. *The ethanol extract of *Dioscorea trifida *was effective against *E. coli, S. aureus*, and *P. aeruginosa. Caesalpinia spinosa* was the only species that showed high activity against all bacteria, including *Salmonella enterica Ttyphi *and* Pseudomonas aeruginosa*, when extracted in ethanol.

Two hundred twenty-five extracts came from plant species that are traditionally employed against bacterial infections. One hundred sixty-six (73.8%) of these were active against at least one bacterium. Of the three hundred extracts from plants without traditional antibacterial use, only 96 (32%) showed any activity. This shows clearly that plants traditionally used as antibacterial had a much higher likelihood to be antibacterially active than plants without traditional anti-bacterial use. However, the efficacy of plants used traditionally for antibacterial related applications did vary, which underlines the need for studies aiming to clearly understand traditional disease concepts. Plants used for respiratory disorders, inflammation/infection, wounds, and diarrhea, and to prevent postpartum infections, were efficacious in 70–88% of the tests. Plants used for “kidney inflammation” had a much lower efficacy against bacteria and fell within the range of species that are traditionally used to treat other bodily disorders. Only species used for spiritual/ritual treatments scored worse. Of these, only 22% showed some antibacterial activity. However, amongst the “spiritual” plants, 38% of the species used for cleansing baths did in fact show activity, while only 15% of the plants often used in protective amulets (mostly species with the families of Lycopodiaceae and Valerianaceae) showed limited antibacterial activity.

A variety of species showed higher efficacy than the control antibiotics employed, for example, *Ambrosia peruviana, Iresine herbstii, Niphogeton dissecta, Opuntia ficus-indica, Smilax kunthii* were particular effective against *Escherichia coli. Berberis buceronis, Caesalpinia paipai, Caesalpinia spinosa, Cestrum strigilatum, Cydista aequinoctialis, Dioscorea trifida, Escallonia pendula, Escobedia grandiflora, Eucalyptus citriodora, Eucalyptus globulus, Eugenia obtusifolia, Eustephia coccinea, Gallesia integrifolia, Geranium sessiliflorum, Hedyosmum racemosum, Iresine herbstii, Lycopersicon hirsutum, Mauria heterophylla, Phyllanthus niruiri, Porophyllum ruderale, Salvia cuspidata, Senecio chionogeton, and Smilax kunthii, Tagetes erecta*, and *Taraxacum officinale* showed high activity against *Staphylococcus aureus*. The same holds true for *Ephedra americana, Gentianella bicolor*, and *Mandevilla *cf.* trianae*. However, extracts of these three species were highly inconsistent in their efficacy.

The comparison of closely related species traditionally employed for different purposes (e.g., different *Alternanthera* spp., *Passiflora* spp., *Senecio* spp., and *Salvia* spp. for spiritual purposes and against bacterial infections) showed that the “spiritual” species normally were not effective against bacteria, while the species used as antibacterials had increased effectiveness. The example of *Plantago sericea* var. *sericea* (used in seguros, no efficacy) and *Plantago sericea* var. *lanuginosa* (used for vaginal infections, high efficacy against *S. aureus*) is a particularly compelling case that indicates the sophistication of traditional knowledge. However, we did find examples like *Chuquiragua* spp., where closely related species were used as antibacterials, but only one of them did in fact show efficacy, clearly indicating that, in this case, traditional knowledge did not produce reliable results.

On the other hand, extracts of the same species traditionally used to treat infections often produced vastly diverging results when collected from different localities. Good examples are *Iresine herbstii*, *Schinus molle*, *Eustephia coccinea*, *Oreopanax eriocephalus*, *Myroxylon balsamum*, *Spartium junceum*, or *Gentianella dianthoides*. Most of these species did not produce particularly high inhibition rates in any case and were not the first choice of healers when trying to find remedies for bacterial infections. Many traditional remedies for concepts like “kidney inflammation” did not produce any antibacterial results, which underlines that research into efficacy does need to closely take traditional disease concepts into account.

Many remedies used for spiritual healing and other noninfection purposes did show antibacterial efficacy *in vitro* but were not listed as such by the local healers. This might be explained by the fact that they either are very inconsistent in their activity (e.g., *Mandevilla trianae*, *Loricaria* spp., *Lonicera japonica*, *Hypericum laricifolium*, *Hyptis sidifolia*, *Mentha piperita*, *Brachyotum naudinii*, and *Cydonia oblonga*) or are so closely related that identification, especially when dried, can be a problem, for example, in the case of *Baccharis* spp., *Gentianella* spp., and *Valeriana* spp., or are prone to toxic side effects like *Ephedra americana* and *Brugmansia* spp.

Almost all remedies are traditionally prepared as water extracts, although ethanol (in the form of sugarcane spirit) is readily available. This might at a first glance seem astonishing, given the low efficacy of water extraction found in this study. However, initial results from Brine-Shrimp toxicity assays indicate that the ethanolic extracts are by far more toxic than water extracts of many species, and thus ethanolic extraction might in many cases not be suitable for application in patients. This again indicates the considerable sophistication and care with which traditional healers in northern Peru chose their remedies for a specific purpose.

If the botanical documentation of Peruvian medicinal plants has been neglected, investigations of the phytochemical composition of useful plants are lagging even further behind. Most studies on the phytochemistry of Peruvian plants concentrate on a few “fashionable” species that have been marketed heavily on a global scale, especially Maca (*Lepidium meyenii*), Sangre del Drago or del Grado (*Croton lechleri*), and Uña de Gato (*Uncaria tomentosa *and* Uncaria guianensis*). The number of other Peruvian plants for which at least some phytochemical studies exist is still miniscule, and most efforts are fuelled by the fads and fashions of the international herbal supplement market. Studies involving multiple species were initiated as late as the 1990s [163].

Minimum inhibitory concentrations found for Peruvian plant extracts ranged from 0.008 to 256 mg/mL. The very high values in many species indicate only a very limited antibacterial efficacy. The ethanolic extracts exhibited stronger activity and a much broader spectrum of action than the water extracts. The most interesting activity on *E. coli* was obtained from ethanolic extracts of *Baccaris *sp., *Ochroma pyramidale*, *Croton lechleri*, *Banisteriopsis caapi*, *Miconia salicifolia*, and *Eugenia obtusifolia*. Only the latter species also showed strong activity in the aqueous extract. A much wider range of species, including most species active against *E. coli* showed inhibition of *S. aureus*. *Porophyllum ruderale, Senecio* sp., *Corynaeae crassa*, *Dioscorea trifida*, *Senna monilifera*, *Spartium junceum*, *Pelargonium odoratissimum*, *Satureja pulchella*, *Cuphea* sp., *Malva parviflora*, *Brosimum rufescens*, *Syzygium aromaticum*, *Sanguisorba minor*, *Citrus limetta*, *Verbesina* sp., and 2 unidentified species all showed MIC values between 1 and 4 mg/mL. Most of them however did not portray any efficacy in aqueous extract. *Hypericum laricifolium*, *Hura crepitans*, *Caesalpinia paipai*, *Cassia fistula*, *Hyptis sidifolia*, *Salvia* sp., *Banisteriopsis caapi*, *Miconia salicifolia* and *Polygonum hydropiperoides* showed the lowest MIC values and would be interesting candidates for future research. Most MIC values reported in this work were largely higher than those obtained for South American species [623–626] and African studies [627]. However, they were in range or lower than concentrations reported by [628, 629].

Most species effective against *S. aureus *are traditionally used to treat wound infection, throat infections, serious inflammations, or are postpartum infections. Interestingly many species used in cleansing baths also showed high activity against this bacterium. Many of these species are either employed topically, or in synergistic mixtures, so that possible toxicity seems not to be an issue. The species effective against *E. coli* were mostly employed in indications that traditional healers identified as “inflammation.”

Most of the plants used by the healers have antibacterial activity, but only 8 of the 141 plants (5.6%) examined in this study show any MIC values of 200 or less mg/mL of extract. Of these 8 plants, 5 are used to treat diseases believed to be in bacterial origin by TM, one is a disease not believed to be caused by bacteria and one is used for undefined treatment purposes.

Nine out of 141 plants (6.3%) tested that were not used for diseases believed to be bacterial in origin by TM, 5 showed high antibacterial activity with MIC values below 16 mg/mL. Four of these were among the most potent plants tested with MIC values of 2 or less mg/mL including the hallucinogen and extracts used to treat diabetes and epilepsy. Diseases such as diabetes often compromise the health of the individual and antibacterial treatments can be warranted for secondary complications of the disease. In addition, TM does determine sometimes that diseases not originally believed to be bacterial in origin, such as ulcers, are actually caused by bacteria. Currently, TM is seriously looking the role of inflammation (which can certainly be bacterial in origin) in heart disease.

### 5.6. Toxicity in Traditional Medicine

Crude medicinal activities have been investigated for a wide variety of plants [86, 131, 132, 136–138, 630–632]. But while toxicity assays are available for many countries (e.g., Argentina [365, 633], Bahrain [634], Bangladesh [635], Brazil [329, 418, 636, 637], Canada [638], Chile [639], China [640], Cuba [641, 642], Ecuador [643], Guatemala [644–646], Honduras [647], India [648], Kenya [627, 649], Mexico [650], Nicaragua [651], Nigeria [652], Panama [653], Papua New Guinea [654], Philippines [655], Uruguay [656], and USA [657–659], no data exists on the potential toxicity of Peruvian medicinal species.

Brine shrimp (*Artemia*) is frequently used as agent in laboratory assays to determine toxicity values by estimating LC_50_ values (median lethal concentration) [651, 660–662]. The Brine shrimp lethality activity of 501 aqueous and ethanolic extracts of 341 plant species belonging to 218 genera of 91 families used in Peruvian traditional medicine was tested [132]. The aqueous extracts of 55 species showed high toxicity values (LC_50_ below 249 *μ*g/mL), 18 species showed median toxicity (LC_50_ 250–499 *μ*g/mL), and 18 low toxicity (LC_50_ 500–1000 *μ*g/mL). The alcoholic extracts proved to be much more toxic: 220 species showed high toxicity values (LC_50_ below 249 *μ*g/mL, with 37 species having toxicity levels of >1 *μ*g/mL), 43 species showed median toxicity (LC_50_ 250–499 *μ*g/mL), and 23 species low toxicity (LC_50_ 500–1000 *μ*g/mL). Over 24% of the aqueous extracts and 76% of the alcoholic extracts showed elevated toxicity levels to brine shrimp. Traditional preparation methods are taking this into account; most remedies are prepared as simple water extracts, thus avoiding potential toxic effects. Excellent examples where the water extracts are nontoxic, while the ethanolic extracts show high toxicity are *Ocimum basilicum* L., *Salvia* sp., or *Laccopetalum giganteum* (Wedd.) Ulbrich. In contrast, *Cinchona officinalis* L. ethanolic extracts were non-toxic, and are traditionally used, while the highly toxic water extract has no traditional use.

Species which showed higher levels of toxicity were *Bejaria aestuans* L., *Erodium cicutarium* (L.) L'Her., *Brachyotum naudinii* Triana, *Miconia salicifolia* (Bonp. ex Naud.) Naud., *Cuscuta foetida* Kunth, *Caesalpinia spinosa* (Molina) Kuntze, and *Phyllactis rigida* (Humb. and Bonpl.) Pers. *Achillea millefolium* L., *Artemisia absinthium* L., and* Eucalyptus globulus* Labill all frequently used as medicinal teas also fall in this group, as do* Lupinus mutabilis* Sweet, and* Illicium verum* Hook. f. Solanaceae (e.g.,* Nicotiana tabacum* L. and *Solanum americanum* Mill.) were proved to be highly toxic, while other species, known to be highly toxic when ingested (e.g., *Datura* sp. and *Brugmansia* spp.) did not show toxicity in Brine Shrimp.

Multiple extracts from different collections of the same species showed in most cases very similar toxicity values. However, in some cases, the toxicity of extracts from different collections of the same species varied from non-toxic to highly toxic. Examples for such variation in toxicity were found for *Chersodoma deltoidea* M.O. Dillon and Sagast., *Satureja sericea* (C. Presl. and Benth.) Briq., *Eugenia obtusifolia* Cambess., *Epidendrum* sp., *Capparis crotonoides* Kunth, *Sambucus peruviana *Kunth, and *Malva *sp. In case of these frequently used species, harvest time, collection locality, or use of specific plant parts might be important for a reduction of toxicity.

Toxicity values with LC_50_ values below 1000 *μ*g/mL are considered to be bioactive and might provide leads for further screening [660]. Over 75% of the species in the present study might have some cytotoxic potential. The toxicity values reported fall in the range reported by other authors [651].

### 5.7. Markets and Sustainability

#### 5.7.1. The Pharmacopoiaa of Southern Ecuador and Northern Peru: Colonial Regimes and Their Influence on Plant Use

The differences in medicinal plant use between Southern Ecuador and Northern Peru are striking. Both regions share the same cultural background and have a very similar flora, with a comparable number of plant species that to a large extent overlap. However, the medicinal flora of Southern Ecuador includes only 40% of the species used in Northern Peru. The differences in traditional medicinal use can be explained by comparing the development of the pharmacopoeia of both areas from the start of the colonial period until today. Colonial chroniclers often included detailed descriptions of useful plants in their reports. The most comprehensive early accounts of the economically interesting flora of Northern Peru and Southern Ecuador were provided by Monardes [15], Acosta [16], and Cobo [17, 18]. Later treatments were included in Alcedo [663]. Martínez Compañon, Archbishop of Trujillo, had a complete inventory of his dioceses prepared [19]. Finally, Ruiz provided the first real botanical inventory of the region [22]. The account of Martínez Compañon [19] provides the best baseline for a comparison of the colonial and modern medicinal flora of the region. The work includes detailed paintings for every species, which allows a close comparison with the modern medicinal flora, indicating that the vernacular names of useful plants have not changed significantly since colonial times. It contains 526 useful plant species. A preliminary review of this work seems to indicate that the number of plants used has not changed significantly since the late 1700's, with over 500 plant species still found in modern Peruvian markets. A closer comparison shows, however, that only 41% of the species mentioned by Breevort [11] are still sold nowadays in Peru. An additional 32% are still used in the Amazon basin but do not reach the coastal markets anymore. Twenty-seven percent have completely disappeared from modern day use. This means that 58% of the species sold in Peruvian markets and 41% of the species used in Ecuador were added to the pharmacopoeia within the last 200 years.

A cluster analysis of the colonial and modern plant inventories showed a striking explanation for the use differences between Ecuador and Peru and helps to explain why the plant inventories changed so significantly in the 18th century. The current pharmacopoeia of useful flora in Ecuador was most similar to the early colonial flora mentioned in Tilbert and Kaptchuk [12], Domenighetti [7], Eisenberg et al. [9, 10], and Zollman and Vickers [8]. This indicates that the Ecuadorian medicinal flora did not develop much between early and late colonial times. In contrast, the modern Peruvian healing flora was much more similar to later collections. An explanation for this lies in the different treatment of traditional practices in Ecuador and Peru. In Ecuador, traditional medicinal practitioners were immediately persecuted once the colonial administration took hold, while the Peruvian administration was much more tolerant. This also reflects in the establishment of a National Institute for Traditional Medicine in Peru in the 1980s, while traditional medicine was illegal in Ecuador, until a constitutional change in 1998. This meant that Ecuadorian healers had no opportunity to experiment with new species to cure diseases introduced by Europeans, while Peruvian healers were able to explore the rich flora of the region in order to find new remedies. This experimentation also extended to “magical” disease concepts like *Mal Aire, Mal Ojo, Susto, *and *Envidia* that were introduced from Spain during the colonial regime. Peruvian healers developed a vast array of medicinals to treat these conditions, which, to a large extent, explains the shift in the medicinal flora between the late 1700s and modern times. Experimentation in Ecuador remained restricted to the treatment of common diseases, while spiritual treatments were outlawed until a constitutional revision in 1998 recognized the right of the population to use traditional medicinal practices [157].

#### 5.7.2. Changing Markets

Exotics played an important role amongst all pants sold in Northern Peruvian markets. Fifty-nine species (15%) found in all markets were exotics. However, amongst the species most commonly encountered in the inventories, 40–50% were exotics. *Matricaria recutita *(chamomile) was found in the inventory of approximately 70% of vendors. The next most popular species sold in these markets included *Equisetum giganteum*, *Phyllanthus urinaria*, *Phyllanthus stipulatus*, *Phyllanthus niruri *(Chanca piedra—stone breaker), *Eucalyptus globulus (eucalyptus), Piper aduncum, Uncaria tomentosa* (cat's claw), *Rosmarinus officinalis* (rosemary)*, Peumus boldus, Bixa orellana* (achiote) and *Buddleja utilis*. However, when taking sales volume into account, *Croton lechleri* (dragon's blood), *Uncaria tomentosa*, and *Eucalyptus globulus* were clearly the most important species [664].

While it was very easy for all vendors to name their most important and frequently sold species, it proofed impossible to get detailed information about species that vendors observed as “rare” or “disappearing”. In most cases, vendors mentioned species as rare because they themselves did not sell them; in many cases, these plants were very common outside the market (e.g., *Plantago major *or common plantain) or because demand was so low, that it would not have made sense to carry them in their inventories. Very small vendors had inventories that represented the most common medicinal plants available and excluded most species in the large “witchcraft” segment of the pharmacopoeia. On the other hand, well-established large stands specialized in supplies for healers (including “magical” plants).

All four markets had inventories containing more than 50% of all inventoried plant species but lacked many of the “generalist” plants sold by other vendors. The portfolio of these stands focused almost entirely on “magical” species that are needed to cure illnesses like “*susto*” (fright), “*mal aire*” (evil wind), “*daño*” (damage), “*envidia*” (envy), and other “magical” or psychosomatic ailments. At the same time, all four vendors catered also to the esoteric tourism crowd that tends to frequent the large markets and carried a variety of plants that were not used by *curanderos* but instead were sold to meet tourist demand.

#### 5.7.3. A Look on Sustainability—How Much Plant and for Which Price?

More than two-thirds of all species sold in Northern Peruvian were claimed to originate from the highlands (*sierra*), above the timberline, which represents areas often heavily used for agriculture and livestock grazing. The overall value of medicinal plants in these markets reaches a staggering 1.2 million US $/year. This figure only represents the share of market vendors and does not include the amount local healers charge for their cure. Thus, medicinal plants contribute significantly to the local economy. Such an immense market raises questions of the sustainability of this trade, especially because the market analysis does not take into account any informal sales.

Most striking was the fact that 7 indigenous and 3 exotic species, that is, 2.5% of all species traded, accounted for more than 40% of the total sales volume (with 30 and 12% resp.). Moreover, 31 native species accounted for 50% of all sales, while only 16 introduced plants contributed to more than a quarter of all material sold. This means that little over 11% of all plants in the market accounted for about three-fourths of all sales. About one-third of this sales volume includes all exotic species traded. None of these are rare or endangered. However, the rising market demand might lead to increased production of these exotics, which in turn could have negative effects on the local flora [127].

A look at the indigenous species traded highlights important conservation threats. *Croton lechleri* (dragon's blood), and *Uncaria tomentosa* (cat's claw) are immensely popular at a local level and each contributes to about 7% to the overall market value. Both species are also widely traded internationally. The latex of *Croton* is harvested by cutting or debarking the whole tree. *Uncaria* is mostly traded as bark, and again the whole plant is normally debarked. *Croton* is a pioneer species, and, apart from *C. lechleri*, a few other species of the genus have found their way in the market. Sustainable production of this genus seems possible, but the process has to be closely monitored, and the current practice does not appear sustainable because most *Croton* is wild harvested. The cat's claw trade is so immense, that in fact years ago collectors of this primary forest liana started complaining about a lack of resources [63] and, during the years of this study, other *Uncaria* species, or even *Acacia* species, have appeared in the market as “cat's claw” (own observation). As such, the* Uncaria* trade is clearly not sustainable.

Some of the other “most important” species are either common weeds (e.g., *Desmodium molliculum*) or have large populations (e.g.,* Equisetum giganteum*). However, a number of species are very vulnerable. *Tillandsia cacticola* grows in small areas of the coast as epiphyte [665]. The habitat, coastal dry forest, and shrub are heavily impacted by urbanization and mechanized agriculture the impact of the latter worsened by the current bio-fuel boom.

*Gentianella alborosea, G. bicolor, G. graminea, Geranium ayavacense*, and *Laccopetalum giganteum* are all high altitude species with very limited distribution. Their large-scale collection is clearly unsustainable, and, in case of *Laccopetalum*, collectors indicate that supply is harder and harder to find. The fate of a number of species with similar habitat requirements raises comparable concern. The only species under cultivation at this point are exotics and a few common indigenous species.

When looking at the reasons why people chose medicinal plants or pharmaceuticals for greater consumption, it seemed as though the major reasons were fairly obvious. Many people preferred using plants more often because they are natural and safe. Pharmaceutical products have too many synthetic chemicals and foreign substances that can affect the body. Using plants that have been in use for centuries seems to be a safer and healthier alternative. Many people said that pharmaceuticals were used for particular illnesses, but often had side effects that result in negative impacts elsewhere in the body. Respondents agreed, however, that pharmaceuticals products were more effective than medicinal plants. Even though they still used plants, they would not completely depend on them, knowing that there is a limit to their use. A lot of agreement was registered for use of doctor's prescriptions. Many people have faith in their doctor, and if he recommends using a certain medicine, they will. This faith is based on the confidence people have in science and medicine with a great deal of research available, which has gained the public's trust. Because of this, people feel safer relying on modern medicine. Along with the research, people know that medicine has noticeable effects that can be more easily obtained than those from plants. Plant remedies take longer and are more subtle in their effects. These are reasons why pharmaceuticals are used more often. Although the number was minimal, there were respondents who did say that they used the two kinds of medicine in the same amounts. What was interesting was that people said that they used both together. For example, often people said that they would drink a cup of herbal tea while taking pills. Although people felt that each type of medicine has a role, most agreed that pharmaceuticals provide the best route taken for fighting certain sicknesses.

## 6. Final Comments

Current research indicates that the composition of the local pharmacopoeia in Northern Peru and Southern Ecuador has changed since colonial times [19, 21, 157]. However, in Northern Peru, the overall number of medicinal plants employed seems to have remained at a comparable level, while plant use in Southern Ecuador has decreased. This indicates that the Northern Peruvian health tradition is still going strong and that the healers and public are constantly experimenting with new remedies. One example of this is the sudden appearance of Noni (*Morinda citrifolia*) fruits and products in large quantities in plant pharmacies and markets in the region since 2005. This plant was not available before, but it is heavily marketed worldwide. Peruvian sellers are clearly reacting to a global market trend and are trying to introduce this new species to their customers. This indicates that local herbalists and herb merchants are carefully watching international health trends to include promising species in their own repertoire. In Southern Ecuador, healers were not able to experiment with new remedies due to persecution and legal restrictions. As a result, the pharmacopoeia in this region remained on an early colonial level, with loss of significant knowledge.

The use of hallucinogens, in particular the *San Pedro* cactus (*Echinopsis pachanoi*), is still a vital component of Andean healing practices and has been around for millennia [125]. *San Pedro* can often be found in Cupisnique and Moche iconography. Five hundred years of suppression of traditional healing practices by Western medicine have not managed to destroy this tradition in Peru. The use of *San Pedro*, together with additives like Angel's Trumpet (*Brugmansia* spp.), Jimsonweed (*Datura ferox*), and tobacco, is still a central part of curing ceremonies in Northern Peru. Healers are in fact experimenting with new hallucinogens, and some northern *curanderos* have started to include decoctions of Ayahuasca (*Banisteriopsis caapi*) in their rituals.

Although not formally acknowledged, Southern Ecuador falls into the Northern Peruvian cultural area. It appears to represent a region where traditional plant knowledge, though important, has declined considerably. Southern Ecuadorian *curanderos* and *parteras* (midwives) have almost entirely abandoned indigenous rituals. In fact, *San Pedro* usage was not mentioned as a mind-altering plant by any healer or midwife interviewed and was not used in curing ceremonies. Centuries of prohibition have led to a pronounced abandonment of traditional knowledge. This is also reflected in the current study. Many plants used for “magical” purposes in Peru [125] have disappeared from traditional use in Ecuador. The fear of prosecution is still very deeply rooted in the healer community, and most healers interviewed stated that they did not wish to be cited by name. Most healing altars or *mesas* in Southern Ecuador are almost entirely devoid of any “pagan” objects such as seashells pre-Columbian ceramics. Patients are cleansed, by spraying them with holy water and perfumes. In rare cases tobacco juice and extracts of Jimson weed (*Datura ferox*) are used to purify the patients. Southern Ecuadorian *mesas* are also much less elaborated than the *mesas* of Peruvian *curanderos*. The incantations used by healers during their curing session center on Christian symbolism. References to Andean cosmology are almost entirely absent, and the use of guinea pigs as diagnostic instruments has all but disappeared from the tool kit of these healers.

Interestingly, Peruvian *curanderos* have started to fill this spiritual void in Southern Ecuador. Healers from the Northern Peruvian mountains and coastal plains frequently cross over to Ecuador to offer their services to patients—including increasing numbers of foreigners with a “New Age” orientation—who are not satisfied with the more Westernized approach of Ecuadorian healers. These Peruvian colleagues have much more elaborate plant knowledge, and their *mesas* as well as their incantations follow a more traditional pattern.

The knowledge of medicinal plants is still taught by word of mouth, with no written record [126]. Illustrated identification guides for the medicinal plants of Northern Peru and Southern Ecuador and their uses [24, 124] will hopefully help to keep the extensive traditional knowledge of this area alive. However, Traditional Medicine is experiencing increasing demand, especially from a Peruvian perspective, as indicated by the fact that the number of herb vendors, in particular in the markets of Trujillo, has increased in recent years. Also, a wide variety of medicinal plants from Northern Peru can be found in the global market. While this trend might help to maintain traditional practices and to give traditional knowledge the respect it deserves, it poses a serious threat, as signs of overharvesting of important species are becoming increasingly apparent.

Today the most serious threat to this millennial tradition is the destruction of medicinal plant habitats. Urban sprawl and the sugar industry have already greatly altered the coastal plains around Trujillo and Chiclayo. Climatic change and deforestation are threatening the mountain forest systems that are the source of many medicinal species. Most importantly, the high Andean ecosystems and sacred lagoons where many medicinally active species are found are in danger of being destroyed by large-scale mining activities [63, 666].

It is apparent that the respondents used medicinal herbs more often than pharmaceutical medicines, but only to a small degree. Bussmann et al. [129, 130] showed in their studies that patients both at western and herbalist clinics often had a preference for pharmaceutical medicines only to a small degree. People generally assumed that plants are healthier and better to use because they are natural and are thought to not have any side-effects. It is difficult to determine if the knowledge of the use of medicinal plants is growing or decreasing, but the indications are that the last generation knows more than the present. However, most of the present generation does teach their children about the use of medicinal plants. The present study also showed what medicinal plants the respondents used for which purposes. It would be interesting to evaluate the properties of the species used in bioassays. Similarly, the plant knowledge of patients at both facilities was largely identical, with an essentially overlapping selection of common, mostly introduced, species, and basically the same number of medicinal plants mentioned overall. This indicates that traditional medicinal knowledge is a major part of a people's culture that is being maintained while patients are also embracing the benefits of western medicine.

This attitude does however lead to profound challenges when it comes to the safety of the plants employed, in particular for applications that require long-term use. Bussmann et al. [667] found that various species were often sold under the same common names. Some of the different fresh species were readily identifiable botanically, but neither the collectors nor the vendors do made a direct distinction between species. However, often material was sold in finely powdered form, which makes the morphological identification of the species in the market impossible and greatly increases the risk for the buyer. The best way to ensure correct identification would be DNA bar-coding. The necessary technical infrastructure is however not available locally. The use of DNA bar-coding as quality control tool to verify species composition of samples on a large scale would require to carefully sample every batch of plant material sold in the market. The volatility of the markets make this is an impossible logistical task. Often the same or closely related species mentioned in literature sell under wide variety of common names. Worse, one species might be sold; for example, “Hercampuri,” in one location or market stand, while selling under a different name at a neighboring stand. As expected there is no consistency in the dosage of plants used nor do vendors agree on possible side effects.

Studies indicate that the plant use in Northern Peru, although footing on a millennial tradition, has changed considerably even during the last decades. Even in case of plant species used for very clearly circumscribed applications, patients run a considerable risk when purchasing their remedies in the local markets, and the possible side effects can be serious. Much more control and a much more stringent identification of the material sold in public markets and entering the global supply chain via Internet sales would be needed.
